# Fused electron deficient semiconducting polymers for air stable electron transport

**DOI:** 10.1038/s41467-018-02852-6

**Published:** 2018-01-29

**Authors:** Ada Onwubiko, Wan Yue, Cameron Jellett, Mingfei Xiao, Hung-Yang Chen, Mahesh Kumar Ravva, David A. Hanifi, Astrid-Caroline Knall, Balaji Purushothaman, Mark Nikolka, Jean-Charles Flores, Alberto Salleo, Jean-Luc Bredas, Henning Sirringhaus, Pascal Hayoz, Iain McCulloch

**Affiliations:** 1 Department of Chemistry, Imperial College London, South Kensington, SW7 2AZ UK; 2 Key Laboratory for Polymeric Composite and Functional Materials of Ministry of Education, School of Materials Science and Material Engineering, Sun Yat-Sen University, 510275 Guangzhou, China; 3 Department of Physics, University of Cambridge, Cambridge, CB2 1TN UK; 4 KSC, King Abdullah University of Science and Technology (KAUST), Thuwal, 23955-6900 Saudi Arabia; 5 Department of Materials Science and Engineering, Stanford University, 476 Lomita Mall, Stanford, CA 94305 USA; 6 BASF Schweiz AG, RAV/BE, Mattenstrasse, 4058 Basel, Switzerland

## Abstract

Conventional semiconducting polymer synthesis typically involves transition metal-mediated coupling reactions that link aromatic units with single bonds along the backbone. Rotation around these bonds contributes to conformational and energetic disorder and therefore potentially limits charge delocalisation, whereas the use of transition metals presents difficulties for sustainability and application in biological environments. Here we show that a simple aldol condensation reaction can prepare polymers where double bonds lock-in a rigid backbone conformation, thus eliminating free rotation along the conjugated backbone. This polymerisation route requires neither organometallic monomers nor transition metal catalysts and offers a reliable design strategy to facilitate delocalisation of frontier molecular orbitals, elimination of energetic disorder arising from rotational torsion and allowing closer interchain electronic coupling. These characteristics are desirable for high charge carrier mobilities. Our polymers with a high electron affinity display long wavelength NIR absorption with air stable electron transport in solution processed organic thin film transistors.

## Abstract (web summary)

Semiconducting polymers are usually prepared by transition metal mediated coupling reactions that cause problems for sustainability and biological applications. Here the authors synthesise fused electron deficient polymers that are air stable and have high electron affinities, via metal free aldol polymerisation reactions.

## Introduction

Within the field of organic semiconductors, there is considerable interest in highly performing polymers for applications such as solar cells, organic light emitting diodes and transistors^1,2^. Conjugated polymers have the potential for solution processability, compatibility with flexible substrates, and can be fabricated in large areas at high throughput. To fully exploit this technology, there is still much effort required to improve the parameters affecting device performance. For transistor applications, polymer molecular conformation and thin film morphology have been shown to influence charge carrier mobility, and subsequently the device electrical properties^3^. Understanding and controlling the molecular features that impart a favourable backbone orientation and facilitate close intermolecular interactions are required to optimise morphology and long-range order for efficient charge transport. Additionally, it is also important to exert control over the frontier energy levels in order to minimise barriers to charge injection and reduce charge trapping, particularly for electron transport where high electron affinity semiconducting polymers are required^4–6^. There are now established molecular motifs that facilitate high charge carrier mobilities in conjugated polymers, including side chain organisation, short contacts and low energetic disorder^3,7^. Central to many of these design approaches is to introduce molecular functionality that can induce dipole−dipole interactions, facilitating close intermolecular packing, short π−π distances, and therefore potentially optimal morphology for transport^8^.

Diketopyrrolopyrrole and isoindigo are two such examples^9–11^. Both comprise an electron-deficient bis-lactam motif, which has been demonstrated to facilitate strong intermolecular interactions through the dipolar carbonyl groups, and subsequently high ambipolar charge carrier mobilities in many of the copolymers containing these units^8,11–13^. Electron transport is attributed to the combination of a delocalised LUMO level along the polymer backbone, and a sufficiently large electron affinity to facilitate electron injection. In addition, a high electron affinity is also required for ambient stability, as it prevents common redox reactions involving water and oxygen which can occur under device operation, and lead to degradation. The electron withdrawing bis-lactam functionality in these structures are responsible for increasing electron affinity and is typically diluted by the requirement for a more electron rich repeat unit to undergo a transition metal-mediated cross coupling polymerisation. Therefore, a design strategy that only incorporates electron-deficient repeat units into the polymer backbone is desirable^14^.

Here we report an aldol polymerisation scheme that circumvents cross coupling and links repeat units with carbon−carbon double bonds, in contrast to the single bond links in all conjugated polymer backbones prepared in the literature to date. This aldol polymerisation enables access to a class of fully fused polymers^15–17^. Motivation for this approach originates from recent work showing that the energetic disorder in conjugated polymers can strongly influence charge transport; we expect therefore that elimination of rotational torsion between conjugated repeat units along the polymer backbone, through removing single bond links, reduces conformational disorder and eliminates this impediment to charge transport. In addition, non-covalent interactions have been shown to enable backbone planarisation in isoindigo polymers leading to high charge transport^18,19^. Our approach aims to combine these strategies^3^.

Five rigid polymers were synthesised using aldol polymerisation conditions. Within this series of polymers, we explored the effect of aromatic size, backbone dihedral angle and alkyl chain density on the charge carrier mobility as measured in transistor devices. Air stable, electron mobilities of up to 0.03 cm^2^ V^−1^ s^−1^ were achieved.

## Results

### Synthesis of rigid polymers

**Fig. 1:**
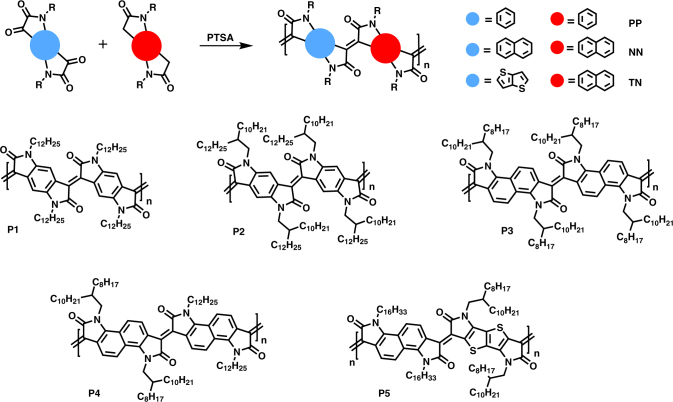
The generic scheme for the preparation of rigid polymers synthesised via aldol polymerisation. The polymers are made from bis-isatin and bis-oxindole monomers. The aldol condensation polymers reported here are P1, P2, P3, P4 and P5. The polymer repeat units are described as PP for phenyl homopolymer, NN for naphthalene homopolymer and TN for thieno[3,2-b]thiophene and naphthalene copolymer. R is a solubilising alkyl chain

The aldol condensation is a useful tool in synthetic organic chemistry for crafting carbon frameworks, particularly in natural product synthesis. Over the years, there has been a steady increase in the introduction of sophisticated catalysts to tackle challenges of regioselectivity, chemoselectivity, diastereoselectivity and enantioselectivity. Here, we capitalise on the traditional Lewis acid catalysed aldol condensation using p-toluene sulphonic acid. The enolisable carbonyl and nucleophilic carbonyl are bis-oxindole and bis-isatin monomers respectively, as illustrated in Fig. 1. These monomers enable bi-directional aldol condensation resulting in rigidification of the subsequent polymer backbone. Polymerisation is expected to proceed without synthetic side reactions or mis-couplings, given the high molecular weights (Mn = >200 kDa) obtainable. Despite this, a model study of the condensation reaction was carried out, confirming the lack of side reactions, as shown in the Supplementary Methods. We also observed no quantifiable side products when isatin and oxindole are subjected to the polymerisation conditions. This model reaction provides a simple template to examine the fidelity of the polymer synthesis via aldol polymerisation. Furthermore, the potential for Michael additions to the polymer backbone were simulated by heating isoindigo with stochiometric oxindole in the polymerisation conditions and in basic conditions with triethyl amine to replicate conditions conducive to Michael additions. In the case of a Michael addition in basic conditions, the oxindole enolate would attack the central double bond in the isoindigo compound and the resulting carbanion would protonate from the triethyl amine conjugate acid. This species would be identifiable in the proton NMR by the upfield shift in the aromatic protons relative to isoindigo due to the loss in conjugation. However, no Michael addition products were observed in either of these reactions. The potential for Michael addition side products is reduced for these bis-isatin and bis-oxindole systems in the acidic polymerisation conditions^20–23^. Michael additions to isatins and oxindole systems generally require basic conditions and stronger nucleophiles than are available during polymerisation. Likewise, sulphonation of the monomers or polymers is likely insignificant due to low electron density^24^. The repeat unit structure also comprises a high density of electron withdrawing lactam groups, advantageous for increasing the electron affinity. A series of very electron-deficient rigid fused polymers have been prepared in good yields and their property−structure relationship examined.

Minor self-condensation of oxindole was observed between the coupling of bisisatin and oxindole in air (Supplementary Methods), resulting in the formation of a small molecule dimer naphthalene-isoindigo, possibly via oxidation of the oxindole to the isatin. It is therefore necessary to conduct the polymerisation under an inert atmosphere to prevent the potential oxidiation of the bisoxindole; this side reaction could cause the continuation of polymerisation propagation, which would lead to stochiometric imbalance of the monomers and limit molecular weight.

The conjugated π−electron systems of our polymer backbones are locked into a fully rigid conformation, with no single bond twisting, thus minimising conformational disorder. A variety of aromatic cores can be incorporated into the polymer backbone providing control of the frontier energy levels, and electronic coupling. In addition, the synthetic approach via a metal-free aldol condensation polymerisation circumvents conventional polymerisation mechanisms such as Suzuki-Miyaura, Stille or Kumada coupling in which the use of transition-metal catalysts is required. Importantly, highly toxic reagents such as organostannannes and the resulting organotin by-products that require careful removal from both a hazardous and electrical impurity perspective are avoided. Our polymerisation route is more environmentally benign, with water as the only by-product, and there is no need for transition metals or their sequestering purification steps.

**Fig. 2:**
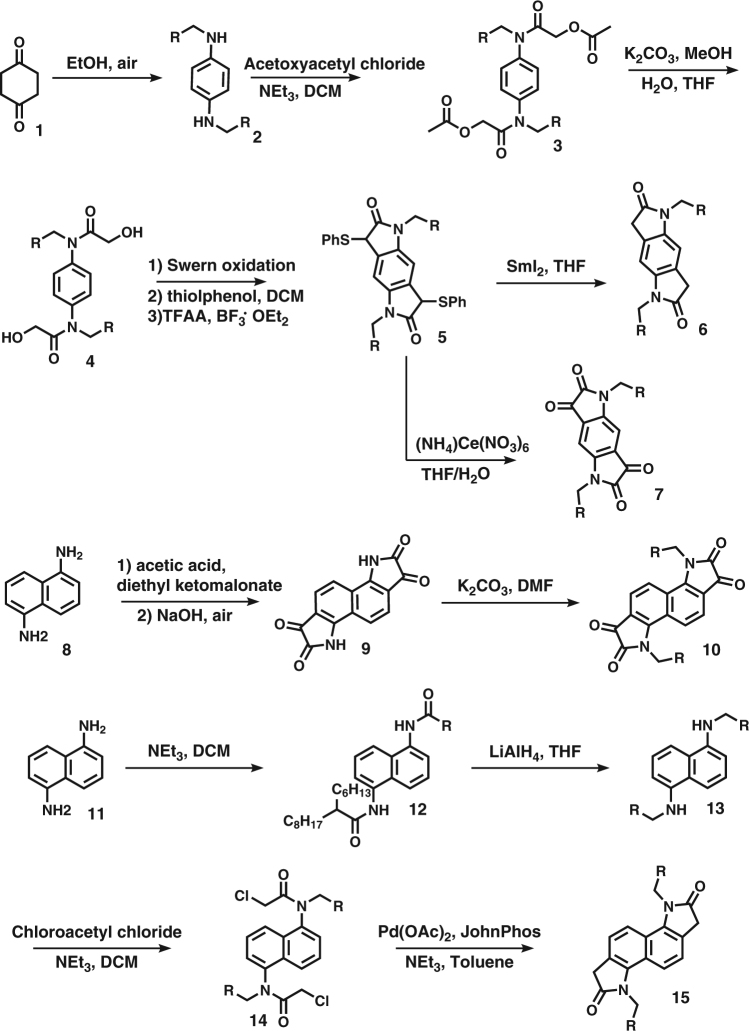
Synthesis of phenyl bis-isatin, phenyl bis-oxindole, naphthalene bis-oxindole and naphthalene bis-isatin monomers. The letter R represents different solubilising alkyl chains used in the synthesis of each monomer as outlined in Supplementary Methods

The synthesis of the phenyl and naphthalene core monomers is shown in Fig. 2. The phenyl and naphthalene bis-isatin and bis-oxindole monomers were synthesised as reported in the literature^25–27^. Preparation of the phenyl bis-isatin and bis-oxindole (Supplementary Methods) begins with oxidation of cyclohexanedione in ethanol, with amination and aromatisation in the same reaction, followed by acylation with acetoxyacetyl chloride and ester hydrolysis to expose two hydroxyl groups. A Swern oxidation of the intermediate dialcohol (4) generates a diglyoxamide prior to a Pummerer-type cyclisation^28^. Oxidation of the resulting tricyclic intermediate (5) produces the phenyl bis-isatin (7), while a reduction furnishes the complementary bis-oxindole (6). The naphthalene bis-isatin is prepared by a Martinet isatin synthesis on 1,5-diaminonaphthalene followed by alkylation with the desired alkyl iodide. Similarly to the naphthalene bis-isatin, synthesis of naphthalene bis-oxindole also starts with 1,5-naphthalenediamine. First, 1,5-naphthalenediamine undergoes acylation with the desired alkanoyl chloride to give naphthalenediamide (12). The diamide is reduced and the resultant diamine reacted with chloroacetyl chloride to give an intermediate (14) suitable for a Heck-type cyclisation to afford the naphthalene bis-oxindole units.

**Fig. 3:**
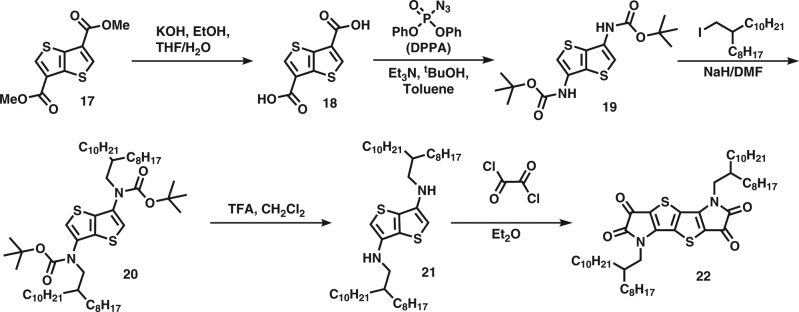
Synthesis of thieno[3,2-b]thiophene bis-isatin monomer. The monomer was synthesised in five steps from dimethyl thieno[3,2-b]thiophene-3,6-dicarboxylate. This synthesis makes use of a Curtius rearrangement reaction to introduce a Boc protected amine to avoid a thieno[3,2-b]thiophene-3,6-diamine intermediate due to their instability

Figure 3 shows the synthesis of the thieno[3,2-b]thiophene bis-isatin monomer. 3,6-dibromothieno[3,2-b]thiophene is lithiated and the dicarboxylate (18) is formed with the addition of solid carbon dioxide. The acyl azide is synthesised and a Curtius rearrangement is carried out in a one-pot reaction in *tert*-butanol to furnish 3,6-bis(N-Boc)thieno[3,2-b]thiophene (19) in good yield. The resulting amino groups in the 3- and 6-position carry Boc (*tert*-butoxycarbonyl) groups allowing alkylation and subsequent deprotection to give the desired 3,6-di(alkylamino)thieno[3,2-b]thiophene (21). Synthesis of the thieno[3,2-b]thiophene bis-isatin monomer by alkylation of 3,6-diamino thieno[3,2-b]thiophene suffers from poor yield, as this substrate is unstable. Nonetheless, treatment with oxalyl chloride in diethyl ether forms the required bis-isatin in poor yields. The amine (21) is rapidly diacylated in the presence of base, the addition of which was found to lower yields, which then undergoes a ring-closing reaction by nucleophilic attack of the ring on the acyl chloride. The main source of low yields is believed to be due to the deactivation of the ring system to the second ring closing step after the completion of the first due to the electron withdrawing effect of the isatin. The polymers were subsequently prepared from the bis-oxindole and bis-isatin in good yields by simply refluxing a toluene solution of the monomers in the presence of p-toluene sulphonic acid monohydrate.

### Characterisation of rigid polymers

We initially selected the phenyl-bis-oxindole and phenyl-bis-isatin monomers with linear dodecyl side chains for the aldol polymerisation. The resultant polymer, P1 (Fig. 1) exhibited a relatively low number-average molecular weight (*M*_n_) of 14 kDa arising from the lack of solubilising alkyl chain density and subsequent precipitation of the growing polymer chain before the degree of polymerisation reached an appreciable level. To obtain a higher molecular weight, larger, branched alkyl chains were required to be incorporated on the polymer backbone. Consequently, branched 2-decyl tetradecyl chains were introduced on both the bis-phenyl-isatin and bis-phenyl-oxindole. In this case the resultant polymer, P2, exhibited a higher *M*_n_ of 58 kDa with a PDI of 2.3 (Table 1), and good solubility in a range of solvents. Since the reorganisation energy and charge carrier mobility are associated with the size and rigidity of the acene, an extended conjugated core length has the potential to lower the reorganisation energy and facilitate charge transport^29^. To further evaluate the influence of the aromatic core, the side chain shape and density on polymer properties, naphthalene bis-oxindole and naphthalene bis-isatin have been synthesised with linear and branched chains. Homopolymers P3 and P4 were also prepared by this condensation method, differing in that P3 has all branched side chains, whereas P4 comprises alternating branched and linear side chains^30^. The lack of alpha C−H bonds on a thieno[3,2-b]thiophene aromatic unit has been shown by density functional theory (DFT) calculations to enable an increased coplanarity along polymer backbones, giving greater π-orbital overlap that can be beneficial for (intra-chain) charge transport properties^31^. Therefore, an alternating copolymer, P5, derived from naphthalene bis-oxindole and thieno[3,2-b]thiophene bis-isatin comonomers was also synthesised. With an exact stoichiometric balance, increasing the reaction time can be used to obtain polymers of longer chain lengths as there is no termination of the polymer reactive end. The acid concentration and subsequent monomer concentration used here allow for monitoring and controlling the molecular weight during polymerisation. For example, the number average molecular weights (*M*_n_) of P2 increased three-fold from 18 to 58 kDa by changing the concentration from 0.03 to 0.055 m (Supplementary Table 2).

**Table 1:** Polymer molecular weight, ionisation energies, electron affinities, optical properties and thin film transistor mobilities

Polymer	*M*_n_ (kDa)	*M*_w_ (kDa)	IP^a^ (eV)	EA^b^ (eV)	*λ*^c^ (nm)	*E*_opt_^d^ (eV)	*μ*_e_ (cm^2^ V^-1^s^−1^)
P1	14	19	5.4	4.4	850	1.01	10^−5^
P2	58	131	5.6	4.5	900	1.10	−
P3	214	677	5.3	4.2	927	1.07	0.0012
P4	134	538	5.2	4.2	927	1.01	0.03
P5	8.3	9.0	5.0	4.2	1128	0.84	0.001

^a^ IP measured by photoelectron spectroscopy in air (PESA)

^b^ EA is crudely estimated by addition of the UV-Vis absorption onset to IP (EA = IP−*E*_opt_), a procedure that neglects the exciton binding energy

^c^ Thin films were spin-cast on glass substrates from chlorobenzene solution, *λ* is the peak of the first low energy absorption band of the polymers

^d^ Estimated optical gap was calculated using onset of the thin-film absorption spectra (*E*_opt_ = 1240/*λ*_onset_)

This operationally simple aldol polymerisation method, successfully synthesised electron-deficient, soluble conjugated polymers without employing any metals and with water as the only by-product. All the polymers exhibited good thermal stability, with a 5% weight loss occurring at just over 400 °C for P1 and above 360 °C for the other polymers as determined by thermogravimetric analysis (TGA). No discernible thermal transitions were observed for any of the polymers when analysed by differential scanning calorimetry between 0 and 350 °C. A strong effect of alkyl side chain length and molecular weight on the polymers solubility was observed. P1 is poorly soluble in common solvents chosen for thin film fabrication, such as chlorobenzene (CB) and ortho-dichlorobenzene (o-DCB) despite the low polymer molecular weight. P2 on the other hand is highly soluble, easily dissolving in hexane, by virtue of the large branched alkyl chain, while P3 is soluble in chlorinated solvents, such as dichloromethane, chloroform and chlorobenzene upon prolonged heating. Although P3 has a higher molecular weight than P4, it is more soluble in chlorinated solvents due to its higher chain density. P4 dissolves only in CB and *o*-DCB due to its very high molecular weight. Although the molecular weight of P5 is low, it lacks solubility in most solvents, other than chlorinated solvents, perhaps due to its more planar backbone and enhanced aggregation.

**Fig. 4:**
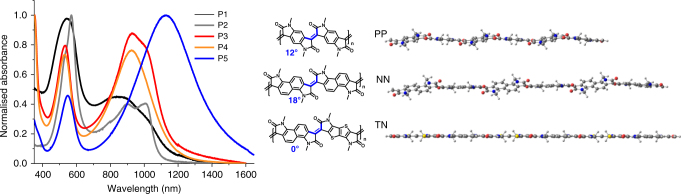
Thin film UV-Vis-NIR absorption spectra and DFT conformational analysis of the polymers. The films were spun from chlorobenzene solutions. P1 and P2 have an all-phenyl core structure (PP), P3 and P4 have an all-naphthalene core and P5 has a thieno[3,2-b]thiophene−naphthalene structure (TN). The molecular geometries were optimised at the ωB97XD/6-31g(d,p) level of theory. The optimal dihedral angles (in degrees) between adjacent aromatic units are shown in blue; the inter-unit double bonds (also highlighted in blue) are 1.37 Å long

Density functional theory calculations were performed to offer an insight into the polymer conformations and frontier energy level features. All geometries were fully optimised at the omega tuned ωB97XD/6-31G(d,p) level of theory for oligomers comprising three repeat units, as shown in Fig. 4. For simplicity, the long alkyl side-chains were replaced by methyl groups. The common feature emerging among the series of oligomers is that, even in the case of isolated chains, the dihedral angles between adjacent aromatic units are very small indeed, on the order of 10–20° in the all-phenylene (PP) and -naphthalene (NN) systems, a feature previously observed for substituted isoindigo^32^, while the thienothiophene-naphthalene (TN) oligomers are fully coplanar; see Fig. 4. The reason for the slight deviations from coplanarity in the PP and NN oligomers is the presence of steric interactions between a carbonyl oxygen and an adjacent C−H group (with an oxygen−hydrogen distance on the order of 2.3 Å). To further explore the conformational implications of steric effects between the fused units, a single-crystal X-ray structure analysis was carried out on a representative dimer of the naphthalene polymers P3 and P4. The synthesis and structure of NIID are given in Supplementary Figure 1. In the solid state, the dihedral angle between the two lactams cores is observed to be 12.8°, again ascribed to short contacts between the carbonyl oxygen and the nearest hydrogen of the adjacent aromatic core, ca. 2.06 Å. These values are in good agreement with the DFT-optimised geometries of the NN oligomer.

The replacement of one of the acene rings with the heteroaromatic thieno[3,2-b]thiophene affords the polymer TN, which removes one of the two adjacent C−H bonds and the related steric interactions, making the coplanar conformation optimal. The DFT potential energy surfaces (PES) calculated as a function of dihedral angle are presented in Supplementary Figure 3. We note that around the minimum, the PES is shallow, which allows for fluctuations in dihedral angles at room temperature on the order of ±10° in isolated oligomers of PP and NN, and ±20° in isolated oligomers of TN (Supplementary Figure 4). This energetically accessible disorder on increasing the dihedral angle is potentially detrimental to planarity, and is in fact similar in energy to more conventional, single bond linked polymers^33^. However, it is expected that such fluctuations will be hampered in the solid state, in view of the tight polymer inter-chain packing discussed below. Also, as we anticipated, the barriers for full rotation are very large, on the order of 25 kcal mol^−1^, which is much higher than the values typical of chains with single-like inter-unit bonds (in the range of 2−8 kcal mol^−1^); this is expected to lead to better chain ordering in the solid state.

The thin film UV-Vis NIR absorption spectra of the polymers are shown in Fig. 4. All polymers exhibit two absorption peaks, with a broad NIR absorption band and a higher-energy band in the visible. Polymers P1 and P2 both show broad and asymmetric long-wavelength absorption peaks extending from around 750 nm to over 1000 nm. P3 and P4 on the other hand have a longer wavelength maximum of 927 nm, due to increased aromatic delocalisation. P5 has the longest wavelength absorption maximum of 1128 nm with a low energy absorption onset of over 1450 nm. The narrower optical gap of 0.84 eV for P5 is due to the combination of a less twisted backbone, and frontier molecular orbital hybridisation between the alternating electron rich thieno[3,2-b]thiophene and electron poor naphthalene lactam. Such low optical gaps are unusual, although not unknown^34^, for typical conjugated aromatic polymers^35–39^.

The vertical excited-state energies were calculated at the time dependent (TD) tuned ωB97XD/6-31g(d,p) level of theory. The absorption spectra calculated for the isolated oligomer chains are presented in Supplementary Figure 5. The energies and strengths of the lowest optical transitions along the PP, NN and TN series closely follow the experimental trends. An analysis of the natural transition orbitals involved in the S_0_- > S_1_ transition, which are illustrated in Supplementary Figure 6, indicates that in all three oligomers both hole and electron wavefunctions are significantly delocalised along the conjugated backbone. The resulting substantial electron-hole spatial overlap leads to large transition dipole moments and oscillator strengths. This finding is consistent with the experimental observation that there is no significant solvatochromic shift (see Supplementary Figure 13), which suggests that these optical transitions do not have a marked charge transfer character. We note that the widths of the low-energy absorption bands are likely to originate in the underlying vibronic progression; and the possible fluctuations in dihedral angles at room temperature (as discussed above). This is consistent with the experimental observation that the broad peaks are not sensitive to thermal treatment, suggesting they are not arising from aggregation. The higher-energy absorptions appearing in the visible also correspond to transitions with significantly delocalised electron-hole wavefunctions; see Supplementary Figure 7.

The reorganisation energies (*λ*) of the PP, NN and TN oligomers were calculated upon injection of an extra electron; the results are collected in Supplementary Table 1 and the anionic geometries and polaron wavefunctions are illustrated in Supplementary Figure 8. The reorganisation energies are in the range of 0.3−0.4 eV with the TN oligomer exhibiting the lowest reorganisation energy in the series, which is mainly due to its maintaining a coplanar conformation even in the presence of the excess electron.

### Electrons transport and stability properties of the rigid polymers

**Fig. 5:**
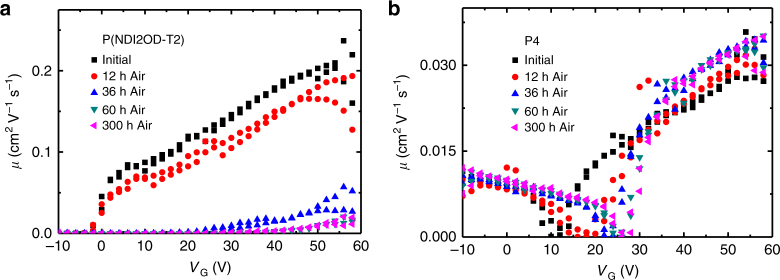
Transistor properties of P4 and N2200. Transistor transfer curves of (**a**) p(NDI2OD-T2)^41^ (N2200) and (**b**) P4 highlighting the air stability of n-charge transport of P4 in comparison to the state of the art p(NDI2OD-T2)

Thin-film transistors with bottom gate top contact architecture were fabricated to investigate the electron transport of the polymers. The charge carrier mobilities were observed to depend on both acene size and the nature of the side chain, and their subsequent influence on intermolecular interactions and therefore on transport. Polymers P1 exhibited low charge transport characteristics, with electron mobility in the range of 10^−5^ cm^2^ V^−1^ s^−1^. Due to the combination of both a small aromatic core as well as high side chain density, shielding intermolecular contacts, P2 showed no charge transport. Increasing the size of the aromatic core from phenyl to naphthyl, in the case of P3, resulted in an improvement in electron mobility in the order of 10^−3^  cm^2^ V^−1^ s^−1^. Previous literature side chain optimisation for fluorene polymer charge carrier mobility has demonstrated the potential advantages of alternating bulky side chains groups with more sterically available segments of the backbone, to register short intermolecular contacts^30^. This approach was exploited in the molecular design of P4 where alternating shorter linear chains and longer bulky branched chains were combined, in comparison to the fully branched polymer P3. This resulted in a large increase in electron mobility with a value of 0.03 cm^2^ V^−1^ s^−1^ obtained. The reduced torsion angle of P5 was also expected to enhance the intermolecular electronic coupling, and thus possibly the transport properties. However, the electron mobility obtained was slightly lower than P4; this could be due to transport being predominately one-dimensional in these polymers, and hence directly related to molecular weight, which in this case was low, because of the low solubility^40^. Interestingly, the large electron affinities of the polymers contribute to excellent ambient stability. Figure 5 compares the transfer characteristics of transistors stored and measured in air for both p(NDI2OD-T2)^41^, an exemplary-type polymer, and P4. The mobility of p(NDI2OD-T2) drops from an initial 0.22 to 0.012 cm^2^ V^−1^ s^−1^ in around 300 h, whereas the mobility of P4 remains relatively stable with a threshold voltage shift from 17 to 27 V.

It has been demonstrated that a deep LUMO is required for n-type electron transport semiconductors to prevent oxidation of the negatively charged polaron in the presence of oxygen^42^. This corresponds to an electron affinity of about 4.2 eV at standard pressures and concentrations. Our polymer design exceeds this threshold, thus eliminating a key electrochemical reaction that can reduce transport in ambient conditions.

**Fig. 6:**
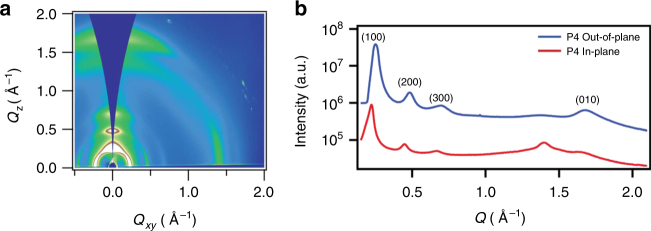
Grazing-incidence wide angle scattering of P4. **a** P4 as cast gives a representative 2D-GIWAXS pattern of some edge-on texture. **b** A horizontal 1D linecut of P4 as cast showing the strong layered ordering (100), (200) and the (010) respectively

Grazing-incidence wide-angle X-ray scattering (GIWAXS) measurements were carried out to gain insight into the polymer microstructure and probe the effect increased conjugation has on molecular packing, as shown in Supplementary Figure 14. Most films exhibit a distinct face-on morphology that appears to be more amorphous in texture, with measured coherence lengths from 1.6 to 2.8 nm. In some cases, morphology was impacted by strong surface temptation due to the limited solubility and reduced film volume displaying strongly oriented face-on texture when compared to bulk measurements. Polymer P4, being the notable exception, which also has significant population of edge-on crystallites that upon annealing coherence lengths increase from 5.5 to 7.1 nm, which garners insight into its enhanced mobility. Figure 6 represents P4 as cast displaying both in-plane and out-of-plane layer stacking peaks. The scattering vector along the intermolecular stacking direction decreases from P1 to P5. The unit cell of these polymers however is complex; therefore, the relationship between interplanar distance and pi−pi stacking distance is not necessarily the same in all compounds. As a result, a firm correlation between the XRD data and the theoretical calculations across molecules in terms of pi−pi stacking distance would only be possible with a more complete understanding of the structure of the unit cell.

## Discussion

Several strategies could be employed to improve polymer properties such as mobility and ambient operational stability. Moving the branch point of the side chain away from the conjugated core is a well-known method to assist inter-chain electronic coupling and as a result, increasing mobilities^43^. Extending the aromatic core or acene could also increase transistor performance. Here, the transport properties were enhanced through extension of the phenyl aromatic core to naphthalene. Further rigid polymer structures fulfilling this design strategy would be central to achieving more competitive charge transport properties. Use of more electron withdrawing aromatic cores such as phenazine would increase the polymer electron affinity and consequently increase operational stability with respect to water and oxygen. Mobility is not an intrinsic value to an organic semiconducting material, it is highly dependent on both device fabrication and measurement method. Future device optimisation will further increase mobilities, by facilitating uniaxially aligned polymer chains through techniques such as blade coating or zone casting^44^, which have been successful in other polymer systems.

In summary, we have outlined a facile synthetic approach to a class of fully fused rigid polymers from a metal-free acid catalysed aldol polymerisation. This polycondensation is the only feasible synthetic strategy to obtaining these fused polymers in metal-free conditions. A series of polymers containing phenyl, naphthalene and thieno[3,2-b]thiophene building motifs with high electron affinities and impressively long wavelength absorption were synthesised. Steric repulsion introduced twists between aromatic cores along the backbones; this however does not hinder crystallisation as shown by the XRD data of the polymers where there are well-defined diffraction features along qxy and qz, and some weaker off-axes reflections. UV-Vis NIR spectra of the polymers show absorption in the near infra-red due to extensive delocalisation of the electron and hole wavefunctions along the polymer backbone. Transistor results demonstrate that air-stable electron mobilities of up to 0.03 cm^2^ V^−1^ s^−1^ can be achieved.

## Methods

### Synthetic methods

All solvents and regents were used as purchased from commercial sources unless otherwise specified. Purifications via flash column chromatography were conducted manually using Merck silica gel (Merc 9385 Kielselgel 60, 230–400 mesh) under positive air pressure with eluent systems as described or on Biotage Isolera.

Proton and carbon NMR spectra were recorded on Bruker DPX0 400 MHz spectrometer. Abbreviations of the peak multiplicity are: bs, broad singlet; s, singlet; d, doublet; t, triplet; q, quartet; m, multiplet, integration and coupling constants (J) are quoted in Hz (hertz) as appropriate. Proton solvent residual peaks are 7.26 ppm for CDCl_3_ and 2.52 ppm DMSO-d_6_. Mass spectra were recorded by the Imperial College London Department of Chemistry Mass Spectrometry Service on a Micromass Autospec Premier/Agilent HP6890 GC. Thermal gravimetric analyses were conducted using Perkin Elmer Pyris 1 TGA.

The polymer molecular weights (number-average *M*_n_ and weight-average *M*_w_) and dispersity were obtained from an Agilent Technologies 1260 series gel permeation chromatography in chlorobenzene at 80 °C. Agilent Technologies Cary Series UV-Vis-NIR spectrometer, Cary 5000, was used for measuring the UV-Vis NIR spectra.

### Grazing-incidence wide angle X-ray diffraction methods

X-ray scattering was conducted at the Stanford Synchrotron Radiation Lightsource at 11-3 (2D grazing-incidence wide-angle with MAR225 image-plate area detector). Incident photon energy was 12.732 keV (0.973 Å). He (g) environments for all measurements were used to minimise air scatter and beam damage to samples. 2D grazing-incidence sample-detector distance was 315 mm calibrated with a polycrystalline lanthanide hexaboride (LaB_6_) standard at a 3.0° angle with respect to the critical angle of the calibrant. For grazing-incidence geometries, the incidence angle was set below the critical angle of 0.1° which is above the critical angle underlying the native oxide substrate. Data was processed using Nika 2D data reduction homebuilt WxDiff Software. The terms *q*_*xy*_ and *q*_*z*_ denote the component of scattering vector in-plane and out-of-plane with the substrate, respectively. Data from 2D grazing-incidence measurements were corrected for the geometric distortion introduced by a flat, plate detector and processed for subsequent analysis with the software WxDiff and GIWAXSTool for Igor^45^. Device parameters were measured on Agilent 4155B Semiconductor Parameter Analyser.

### DFT calculations methods

All geometries were fully optimised at the tuned ωB97XD/6–31G(d,p) level of theory without any constraints. The long alkyl side-chains were replaced by methyl groups to reduce computational costs. All calculations were carried out with the Gaussian-09 software^46^. The range-separation parameter (*ω*) was optimised for each oligomer chain using the gap-tuning method^47^; the optimal *ω* values for PP, NN and TN are 0.104, 0.100 and 0.087 bohr^−1^, respectively.

The reorganisation energy (*λ*) is calculated using following equation.

$${\mathrm{\lambda }} = E_{{\rm nA}} - E_{{\rm nN}} + E_{{\rm aN}} - E_{{\rm aA}},$$

where *E*_nN_ [*E*_aA_] is the total energy of the neutral [anionic] molecule in its optimal neutral [anionic] state geometry and *E*_nA_ [*E*_aN_] is the total energy of the neutral [anionic] molecule in the optimised anionic [neutral] geometry. All calculations were carried out at the OT-ωB97XD/6–31G(d,p) level of theory.

Excited-state energies were calculated at the TD-DFT OT-ωB97XD/6-31G(d,p) level of theory using the ground-state (S_0_) geometries obtained at the OT-ωB97XD/6-31G(d,p) level of theory. Natural transition orbital analyses were also carried out.

### Data availability

The authors declare that the data supporting the findings of this study are available within the paper and its Supplementary Information files. Additional data are available from the corresponding author upon reasonable request.

## Electronic supplementary material

Supplementary Information
