# Supplementary material for: Fused electron deficient semiconducting polymers for air stable electron transport
Source: Nat Commun. 2018 Jan 29;9:416. doi: 10.1038/s41467-018-02852-6 (PMC5789062; doi:10.1038/s41467-018-02852-6)
Supplement: Supplementary file 1 — Supplementary Information [file 41467_2018_2852_MOESM1_ESM.pdf]

Supplementary Methods

Investigating Synthetic Purity through Model Reactions

Significant side products were not observed during the isoindigo condensation reaction, nor could we confirm the presence of the aldol condensation reaction product from the addition of oxindole to isoindigo, under the aldol polymerisation condition of basic aldol condensation medium.

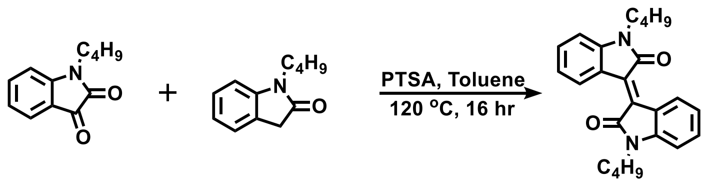

1-butylindoline-2,3-dione (500 mg, 2.46 mmol), 1-butylindolin-2-one (466 mg, 2.46 mmol) were added to a microwave vial along with PTSA (129 L) and the vial sealed. The vial was evacuated and charged with argon three times, prior to the addition of anhydrous toluene (20 mL), degassed for 1 hour and heated at 120 °C for 16 hours. ^{1}H NMR (400 MHz, Chloroform-d) δ 9.18 (d, J = 8.0 Hz, 2H), 7.35 (t, J = 7.7 Hz, 2H), 7.05 (t, J = 7.8 Hz, 2H), 6.80 (d, J = 7.7 Hz, 2H), 3.78 (t, J = 7.3 Hz, 4H), 1.69 (p, J = 7.7 Hz, 4H), 1.43 (q, J = 7.6 Hz, 4H), 0.97 (dd, J = 8.4, 6.0 Hz, 6H). ^{13}C NMR (*101 MHz, $CDCl_3$, 300K*) δ 165.8, 1.42.0, 134.2, 132.4, 130.7, 128.5, 127.6, 126.1, 125.4, 121.1, 120.5, 107.2, 41.8,

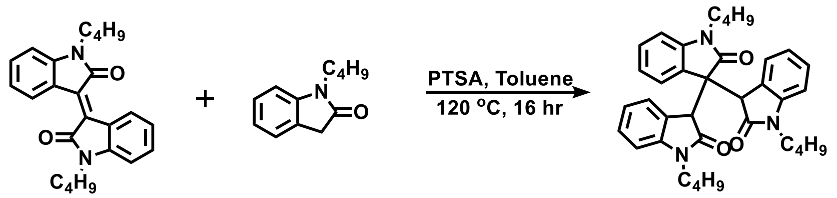

A microwave vial was charged with (*E*)-1,1'-dibutyl-[3,3'-biindolinylidene]-2,2'-dione (300 mg, 0.80 mmol), 1-butylindolin-2-one (152 mg,0.80 mmol) and PTSA (46 mg, 0.24 mmol). The vial was sealed, evacuated and charged with argon three times, before anhydrous toluene (6 mL) was injected into the flask, degassed for 1 hour and heated at 120 °C for 16 hours. The Michael addition product was not observed by proton NMR or LC-MS, only starting materials were observed.

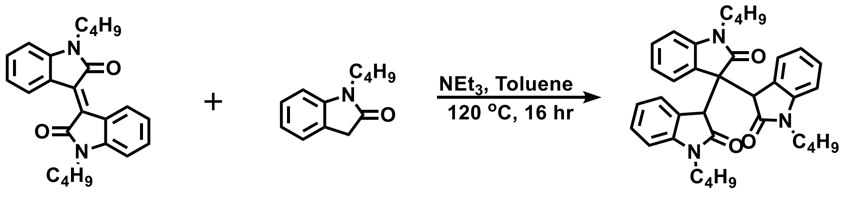

A microwave vial was charged with (*E*)-1,1'-dibutyl-[3,3'-biindolinylidene]-2,2'-dione (300 mg, 0.80 mmol), 1-butylindolin-2-one (152 mg, 0.80 mmol) and triethyl amine (0.03 mL,0.24 mmol). The vial was sealed, evacuated and charged with argon three times, before anhydrous toluene (6 mL) was injected into the flask, degassed for 1 hour and heated at 120 ^{o}C for 16 hours. The Michael addition product above was not observed by proton NMR or LC-MS, only starting materials were observed.

NIID Synthesis and Crystal Data

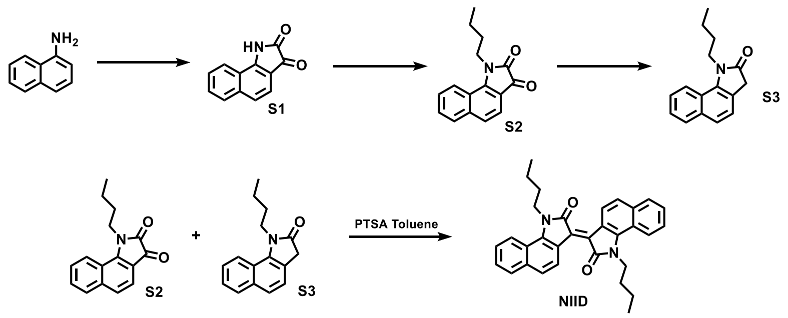

Supplementary Figure 1: Synthetic path to NIID.

Compound S**1** was obtained according to literature procedure.[2]

Compound S**1**: 5-aminonaphthalene (1.82 g, 12.7 mmol) was dissolved in 20 mL of glacial acetic acid and heated to reflux. To the resulting purple solution was added a solution of diethyl ketomalonate (4.0 mL, 26 mmol) in glacial acetic acid (20 mL) dropwise over 30 minutes. The resulting suspension was heated at refluxed for 18 hours. The acetic acid was removed in vacuo and the resulting red solid was dissolved in 1 M NaOH to a final solution with pH of 11-12. The resulting yellow suspension was heated at refluxed with sparging air for 5 hours. The solution was then poured onto ice and acidified to pH 0 with aqueous 6 M HCl. The resulting bright red solid was collected by suction filtration, washed with water and dried in vacuum to yield the crude product as a red solid (1.6 g, 64%). The crude product was used without further purification.

Compound S**2**: In an oven dried flask, crude S**1** (0.8 g, 5.6 mmol) and anhydrous potassium carbonate (1.16 g, 8.4 mmol) were dissolved in 10 mL of dry DMF. The reaction mixture was heated to 50 ^{o}C, 1-iodo-butane (1.27 mL, 11.2 mmol) was added via syringe, and the temperature was kept at 50 for a further 5 hours. The reaction mixture was poured over 100 mL H_2O. The aqueous layer was extracted with chloroform. The organic layers were dried over $MgSO_4$ and concentrated to yield the crude product. The crude product was purified by column chromatography on silica gel (eluent: DCM: hexanes = 1:1), yield 670 mg (47 %). *^{1}H NMR (400 MHz, $CDCl_3$, 300K): δ= 8.21 (d, J = 9.4 Hz, 1H), 7.88 (d, J = 8.9 Hz, 1H), 7.74 –*

7.64 (m, 1H), 7.63-7.59 (m, 2H), 7.57-7.53 (m, 1H), 4.29 – 4.25 (m, 2H), 1.91-1.83 (m, 2H), 1.58-1.48 (m, 2H), 1.01-1.05 (m, 3H).

Compound S**3**: Hydrazine hydrate (0.3 mL) was added to the solution of S**2** (100 mg, 0.40mmol) in 1 mL DMSO, the reaction was kept at 120 ^{o}C for 18 hours. After cooling, H_2O was added, and the mixture was subsequently extracted with ethyl acetate. Organic fractions were combined and washed with brine and dried over $MgSO_4$ to give colourless crude and used without further purification. *^{1}H NMR (400 MHz, $CDCl_3$, 300K): δ= 8.20 (d, J = 7.3 Hz, 1H), 7.91 (d, J = 7.7 Hz, 1H), 7.62-7.59 (m, 1H), 7.53-7.43 (2m, H), 7.43-7.40 (m, 1H), 4.38 – 4.27 (m, 2H), 3.70-3.69 (m, 2H), 1.05-1.00 (m, 3H).*

NIID: Compound S**2** (60 mg, 0.24 mmol) and compound S**3** (80 mg, 0.33 mmol) and PTSA (20 mg, 0.11 mmol) were dissolved in toluene (2 mL) under nitrogen and refluxed for 1 hour. After the reaction was cooled down, the residue was purified by column chromatography (PE: DCM=1:1) to furnish the green product: 85mg (76%). *^{1}H NMR (400 MHz, $CDCl_3$, 300K): δ= 8.92 (d, J = 8.9 Hz, 2H), 8.19 (d, J = 8.6 Hz, 2H), 7.84 – 7.81 (m, 2H), 7.44-7.55 (m, 6H), 4.36 – 4.32 (m, 4H), 1.93-1.85 (m, 2H), 1.58 – 1.50 (m, 2H), 1.36 – 1.28 (m, 2H), 1.01 – 1.05 (m, 3H). MALDI TOF: $C_{32}H_{30}N_2O_2$, Calculated: 474.230, found: 474.175.*

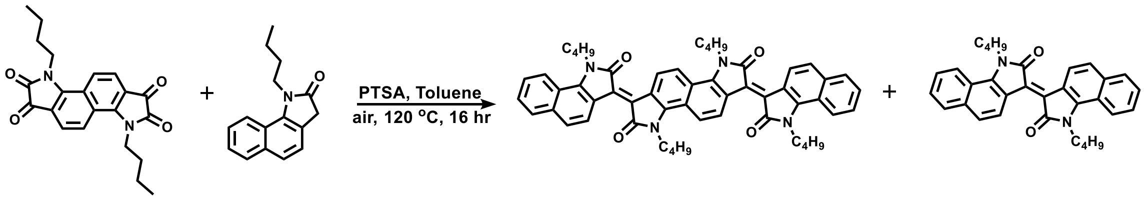

Naphthalene bisisatin-C4 (60 mg, 0.16 mmol) and oxinole-C4 (130 mg, 0.54 mmol) and PTSA (9 mg, 0.05 mmol) were dissolved in toluene (2 mL) under ambient conditions and refluxed for 1 hour. After the reaction was cooled down, the residue was purified by column chromatography (PE: DCM=1:1) to furnish the small molecule (green) and naphthalene isoindigo (cyan).

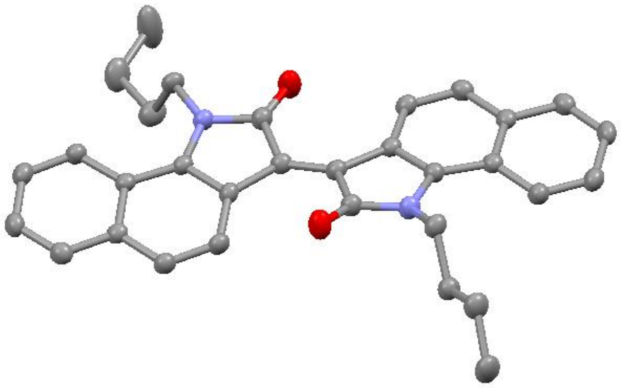

Supplementary Figure 2: Single crystal of NIID.

Here is the single crystal structure of NIID. The dihedral angle of the two lactams is 12.8°. Crystal data: $C_{32}H_{30}N_2O_2$, triclinic P-1, *a*: 15.3052(5) Å, *b*: 15.3815(4) Å, *c*: 21.1879(6) Å, *α*: 89.021(2)°, *β*: 82.798(2)°, *γ*: 76.127(2)°, cell volume: 4803.8(2) Å3, REM R1_all = 0.0974, REM R1_gt = 0.0845, REM wR_ref = 0.2444, REM GOOF = 1.063

Synthesis of Phenyl Core Monomers

The synthetic procedure for the phenyl core monomers were adapted from literature.[1]

Synthesis of *N*,*N*'-bis(2-decyltetradecyl)benzene-1,4-diamine

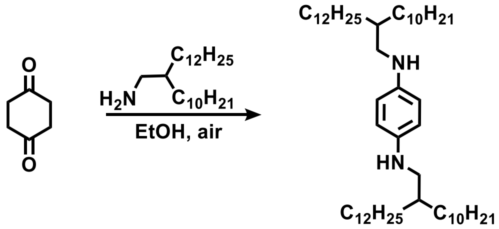

1,4-Cyclohexanedione (0.58 g, 5.0 mmol) was dissolved in 50 mL ethanol and 2-decyltetradecan-1-amine (3.52 g, 9.96 mmol) added to the solution. Air was bubbled through the solution for 2 hours before the solvent was removed under reduced pressure. The red residue was purified on basified silica gel (EtOAc:PE = 3:100) to furnish the title compound (1.8 g, 46 %) as brown oil. *^{1}H NMR (400 MHz, $CDCl_3$, 300K): δ= 6.54 (s, 4H), 3.24 – 2.71 (m, 4H), 1.57 (s, 2H), 1.26 (s, 80H), 0.88 (t, J = 6.7 Hz, 12H). ^{13}C NMR (101 MHz, $CDCl_3$, 300K): δ= 141.18, 114.77, 49.07, 37.95, 32.33, 32.09, 30.25, 29.82, 29.52, 26.90, 22.85, 14.27. MS TOF ES+: $C_{54}H_{104}N_2$, calculated 781.8278, $[M+H]^+$, found 781.8269*.

Synthesis of benzene-1,4-diylbis{[(2-decyltetradecyl)imino]-2-oxoethane-2,1-diydiacetate

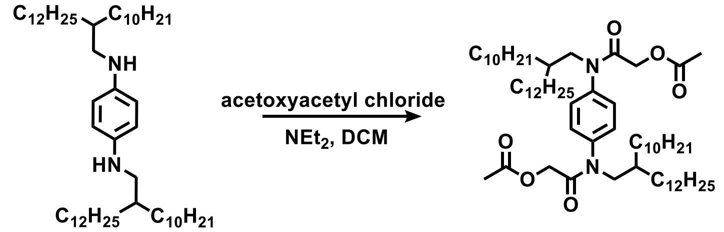

Triethylamine (0.70 mL, 4.99 mmol) was added to *N*,*N*'-bis(2-decyltetradecyl)benzene-1,4-diamine (1.77 g, 2.27 mmol) dissolved in anhydrous DCM (22.70 mL) at 0 ^{o}C. Acetoxyacetyl chloride (0.54 mL, 4.99 mmol) was injected drop wise into the flask before the reaction was warmed to room temperature and stirred for 16 hours. The reaction was quenched with $NaHCO_3$ and EtOAc added. The phases were separated and the aqueous phase extracted three times with EtOAc. The combined organic phase was washed with brine dried over $MgSO_4$, the salts filtered and the solvent removed under pressure to give pale yellow solid, (2.0 g, 96 %). *^{1}H NMR (400 MHz, $CDCl_3$, 300K): δ= 7.32 (s, 4H), 4.33 (s, 4H), 3.64 (d, J = 7.0 Hz, 4H), 2.12 (s, 6H), 1.53 – 1.41 (m, 2H), 1.37 – 1.02 (m, 80H), 0.94 – 0.79 (m, 12H). ^{13}C NMR (101 MHz, $CDCl_3$, 300K): δ= 170.61, 166.56, 141.13, 129.79, 61.79, 53.41, 36.25, 32.06, 31.23, 30.15, 29.82, 29.75, 29.50, 26.39, 22.83, 20.65, 14.25. MS TOF LD+: $C_{62}H_{112}N_2O_6$ $[M+H]^+$: found 980.9.*

Synthesis of *N*,*N*'-benzene-1,4-diylbis[*N*-(2-decyltetradecyl)-2-hydroxyacetamide]

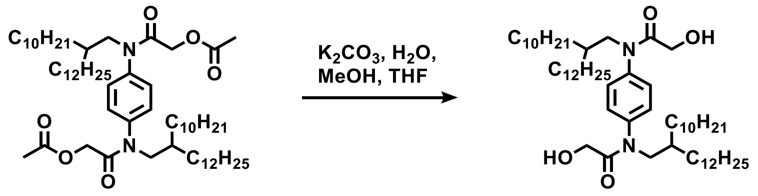

Benzene-1,4-diylbis{[(2-decyltetradecyl)imino]-2-oxoethane-2,1-diyl} diacetate (3.60 g, 3.70 mmol) in THF (200 mL) and MeOH/water mixture (180 ml, 20 mL). The reaction mixture was stirred in the presence of excess K_2CO_3 (5.20 g, 37.0 mmol) at room temperature for 4 hours before the salts were filtered off. The mixture was concentrated under reduced pressure and water and ethyl acetate added to the residue. The phases were separated and the aqueous phase is extracted three times with ethyl acetate. The combined organic phases were washed with brine dried over $MgSO_4$, filtered and the solvent removed in the rotary evaporator to furnish light yellow, 3.1 g, 94%. *^{1}H NMR (400 MHz, $CDCl_3$, 300K): δ= 7.24 (s, 4H), 3.76 (s, 4H), 3.70 (d, J = 7.1 Hz, 4H), 3.47 – 3.30 (m, 2H), 1.52 – 1.37 (m, 2H), 1.36 – 1.04 (m, 80H), 0.86 (t, J = 6.7 Hz, 12H). ^{13}C NMR (101 MHz, $CDCl_3$, 300K): δ= 171.98, 140.31, 129.69, 60.73, 53.47, 36.21, 32.04, 31.23, 30.11, 29.77, 29.71, 29.47, 26.38, 22.80, 14.22. MS TOF LD+: $C_{58}H_{108}N_2O_4$, $[M+H]^+$: found 898.00.*

Synthesis of N,N'-(1,4-phenylene)bis(N-(2-decyltetradecyl)-2-oxoacetamide)

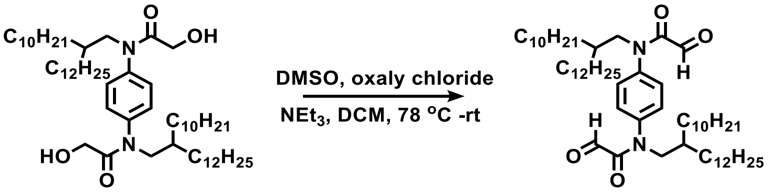

Under argon atmosphere, oxalyl chloride (0.31 mL, 3.89 mmol) was diluted with DCM (4 ml) and cooled to -78 °C. A solution of DMSO (0.28 mL) in DCM (4.2 mL) was added to the reaction flask at -78 °C. The reaction flask was stirred for 20 minutes before a solution of *N*,*N*'-benzene-1,4-diylbis[*N*-(2-decyltetradecyl)-2-hydroxyacetamide] (1.45 g, 1.62 mmol) in 7 mL DCM was injected drop wise into the flask. The reaction mixture turns aqua green. After 1.5 hours at -78 °C, trimethylamine (2.26 mL, 16.2 mmol) was added slowly. The reaction was then stirred at -78 °C for 4 hours before it was warmed to room temperature slowly. The reaction was quenched with saturated $NaHCO_3$ solution and the phases separated. The aqueous phase was extracted three times with DCM and the combined phases washed with brine, dried over $MgSO_4$, filtered and the solvent removed under vacuum to yield brown oil, 0.61 g, which was used immediately.

Synthesis of 1,5-bis(2-decyltetradecyl)-3,7-bis(phenylthio)-5,7-dihydropyrrolo[2,3-f]indole-2,6(1H,3H)-dione

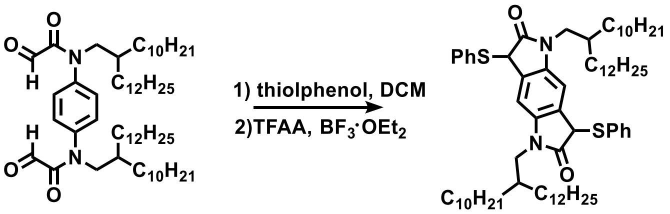

Crude N,N'-(1,4-phenylene)bis(N-(2-decyltetradecyl)-2-oxoacetamide) (1.44 g, 1.61 mmol) was diluted with DCM (6 mL) before thiophenol (0.33 mL, 3.23 mml) was added to flask. The reaction mixture was then stirred for 16 hours at room temperature. Following this, TFAA (2.01 mL, 14.50 mmol) was added slowly to the reaction and stirred for 1 hour 30 minutes, after which, $BF_3 \cdot Et_2O$ (0.99 mL, 8.05 mmol) was added to the flask cautiously. Following further stirring for 3 hours, the reaction was cooled to 0 °C before it was quenched with $NaHCO_3$. The aqueous phase was extracted with DCM three times and the organic phases combined and washed with brine and dried over $MgSO_4$. The solvent was removed under reduced pressure to furnish red /brown residue as the crude product, which was used without further purification. Yield (1.32 g, 76%) *MS (TOF ES+): $C_{70}H_{112}N_2O_2S_2$, calculated 1077.8244, $[M+H]^+$: found 1077.8278.*

Synthesis of 1,5-bis(2-decyltetradecyl)-1,5-dihydropyrrolo[2,3-f]indole-2,3,6,7-tetraone

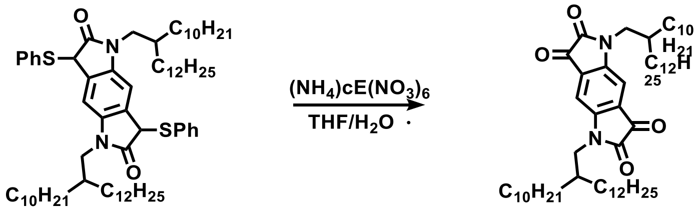

Ceric ammonium nitrate (9.48 g, 17.8 mmol) was added to the solution of 1,5-bis(2-decyltetradecyl)-3,7-bis(phenylthio)-5,7-dihydropyrrolo[2,3-f]indole-2,6(1H,3H)-dione (2.40 g, 2.22 mmol) dissolved in a 6:1 ratio of THF/water (42 mL) mixture. Following 30 minutes stirring at room temperature the reaction mixture takes a deep purple colouration. After 3 hours stirring the reaction mixture was reduced under vacuum. The crude residue was purified by column chromatography on Biotage Isolera at a gradient of 3-10 % ethyl acetate in petroleum spirit 40-60 °C to furnish the titled compound, yield: 300 mg, 15 %. *^{1}H NMR (400 MHz, $CDCl_3$, 300K): δ= 7.12 (s, ArH, 2H), 3.62 (d, J = 7.5 Hz, NCH_2, 4H), 1.84 (d, J = 9.9 Hz, CH, 2H), 1.40 – 1.12 (m, CH_2, 80H), 0.88 (t, J = 6.7 Hz, CH_3, 12H). ^{13}C NMR (101 MHz, $CDCl_3$, 300K): δ= 183.36, 157.15, 147.85, 123.24, 106.99, 77.16, 45.49, 36.15, 32.06, 31.52, 30.12, 29.79, 29.76, 29.69, 29.47, 26.40, 22.82, 14.25. MS TOF LD+: $C_{58}H_{100}N_2O_4$, calculated, 888.77, $[M+H]^+$ found 890.0.*

Synthesis of 1,5-bis(2-decyltetradecyl)-5,7-dihydropyrrolo[2,3-*f*]indole-2,6(1*H*,3*H*)-dione

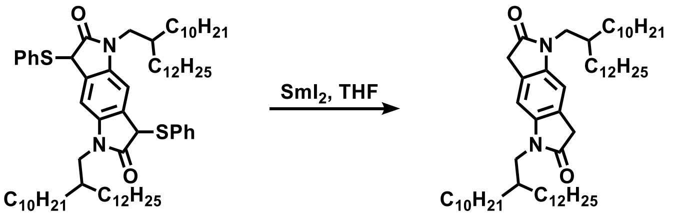

1,5-bis(2-decyltetradecyl)-3,7-bis(phenylthio)-5,7-dihydropyrrolo[2,3-f]indole-2,6(1H,3H)-dione was dissolved in dry THF (37.0 mL) and 0.1 M SmI_2 in THF (40.0 mL, 4.0 mol) added to the solution at room temperature. Following 16 hours, saturated $NaHCO_3$ (200 mL) was introduced into the reaction mixture and the aqueous phase extracted with ethyl acetate three times. The organic layer was washed with brine, dried over $MgSO_4$, filtered and the solvent removed under reduced pressure. Purification by column chromatography in 15 % ethyl acetate in 40-60 °C petroleum spirit afforded 800 mg beige solid; yield: 28 %. *^{1}H NMR (400 MHz, $CDCl_3$, 300K): δ= 6.73 (s, 2H), 3.56 (d, J = 7.9 Hz, ArH 4H), 3.54 (s, CH_2, 4H), 1.91 – 1.76 (m, CH, 2H), 1.24 (s, CH_2, 80H), 0.87 (t, CH_3, J = 6.7 Hz, 12H). ^{13}C NMR (101 MHz, $CDCl_3$, 300K): δ= 174.85, 140.24, 123.92, 106.05, 44.85, 36.41, 36.25, 32.06, 31.68, 30.20, 29.81, 29.49, 26.60, 22.83, 14.26. MS TOF LD+: $C_{58}H_{104}N_2O_2$, calculated, 860.8, $[M+H]^+$ found 860.9.*

Synthesis of Naphthalene Core Monomers

Synthesis of 3,8-dihydroindolo[7,6-*g*]indole-1,2,6,7-tetrone

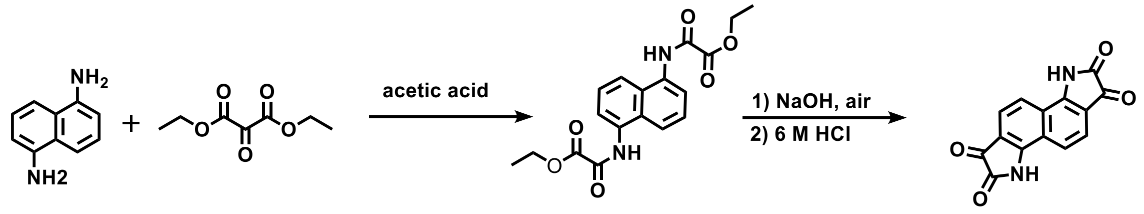

The naphthalene bis-isatin was synthesized according to literature and used without further purification.[2]

Synthesis of 3,8-bis(2-octyldodecyl)-3,8-dihydroindolo[7,6-g]indole-1,2,6,7-tetraone

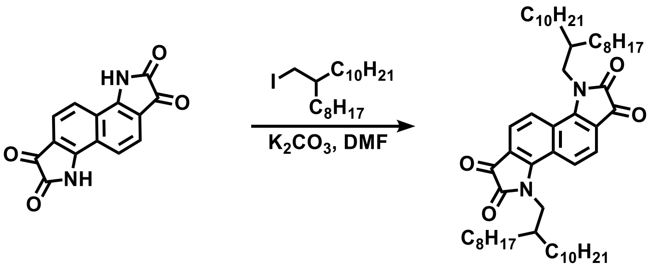

The dark naphthalene 3,8-dihydroindolo[7,6-*g*]indole-1,2,6,7-tetrone solid (1 g, 3.76 mmol), dried K_2CO_3 (4.56 g, 11.26 mmol) were suspended in 10 mL dry DMF and heated to 80 ^oC. The reaction was cooled to 60 ^oC before 1-iodo-2-octyldodecane (2.08 mL, 15.04 mmol) was injected into the reaction. The reaction was heated for 3 hours at 60 ^oC. The reaction was quenched by pouring it into water and acidified to pH 7 with aqueous 1 M HCl. The aqueous phase was extracted with DCM dried over $MgSO_4$ and the solvent removed. The brown residue was purified by flash chromatography with 2:3 DCM/ petroleum spirit 40-60 ^oC, followed by recrystallization in hexane to give blue solid (388 mg, 12.5 % yield). *^1H NMR (400 MHz, $CDCl_3$, 300K): δ= 8.01 (d, J = 8.7 Hz, 2H), 7.68 (d, J = 8.7 Hz, 2H), 4.19-4.17(m, 4H), 1.87-2.01(m, 2H), 1.37-1.24(m, 64H), 0.90-0.87 (m, 12H). ^{13}C NMR (101 MHz, $CDCl_3$, 300K): δ= 182.70, 159.30, 152.39, 127.42, 120.08, 119.94, 116.35, 47.62, 31.09, 29.95, 29.59, 29.52, 26.20, 22.67, 22.65, 14.10. MS TOF LD+: $C_{54}H_{86}N_2O_4$, calculated, 826.66, $[M+H]^+$ found 827.3.*

Synthesis of 3,8-dihexadecyl-1,3,6,8-tetrahydroindolo[7,6-g]indole-2,7-dione

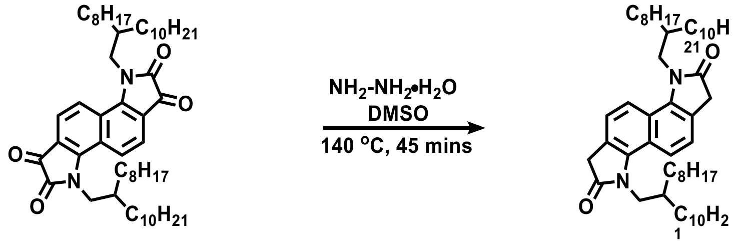

3,8-bis(2-octyldodecyl)-3,8-dihydroindolo[7,6-g]indole-1,2,6,7-tetraone (150 mg, 0.18 mmol) was suspended in 1 ml DMSO and hydrazine hydrate (0.5 ml) and the mixture refluxed for 3 hours. The orange reaction mixture was cooled to 0 °C, and 1 M HCl (3 ml) added to the reaction mixture. The aqueous phase was then extracted with DCM three times. The combined organic phase was washed with brine, dried with $MgSO_4$, filtered and the solvent reduced under reduced pressure. The dark orange crude material was purified by column chromatography on Isolera Biotage with a gradient of 10-40 % ethyl acetate and petroleum spirit 40-60 °C. The resulting material was recrystalised from ethyl acetate to give beige solid (45 mg, 31 % yield). ^{1}H NMR (400 MHz,$CDCl_3$, 300K): δ= 7.96 (d, *J* = 8.6 Hz, 2H), 7.38 (d, *J* = 8.5 Hz, 2H), 4.17 (d, *J* = 7.3 Hz, 4H), 3.68 (s, 4H), 2.11 – 1.86 (m, 2H), 1.44 – 1.30 (m, 13H), 1.28 (s, 5H), 0.87 (td, *J* = 6.8, 3.5 Hz, 12H). ^{13}C NMR (101 MHz, $CDCl_3$, 300K): δ= 177.03, 140.83, 122.05, 121.75, 120.43, 116.40, 47.10, 37.65, 36.15, 32.05, 32.02, 31.35, 30.19, 29.76, 29.71, 29.66, 29.49, 29.42, 26.45, 22.82, 14.26. *MS TOF LD+: calculated, $C_{54}H_{90}N_2O_2$, 798.70, $[M+H]^+$ found 800.9.*

The naphthalene bis-oxindole was synthesized according to literature.[3]

Synthesis of *N*,*N*'-(naphthalene-1,5-diyl)dihexadecanamide

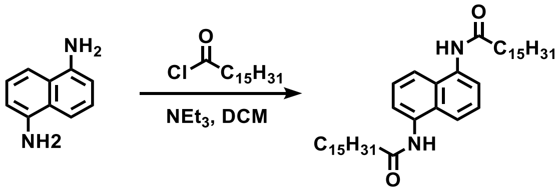

1,5-Diaminonaphthalene (3.0 g, 18.96 mmol) was dissolved in DCM (300 mL) and cooled to 0 °C, triethylamine (0.3 mL, 45.51 mmol) was added to the flask followed by the hexadecanoyl chloride (12.7 mL, 41.71 mmol). The reaction was stirred for 16 hours. The poorly soluble product(12 g, 100 % yield) was filtered and dried in an oven under reduced pressure and used in the next step.

Synthesis of *N*[1],*N*[5]-dihexadecylnaphthalene-1,5-diamine

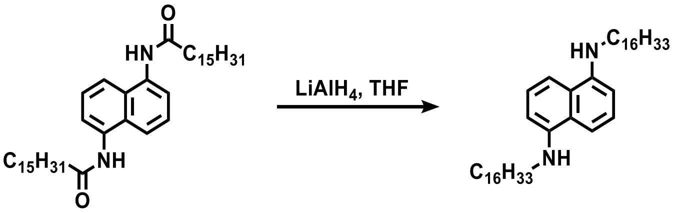

The *N*,*N*'-(naphthalene-1,5-diyl)dihexadecanamide (12.0 g, 18.90 mmol) was suspended in dry THF (160 mL) and cooled to 0 °C. 1 M lithium aluminium hydride (75.6 mL, 75.6 mmol) was added cautiously to the reaction flask and the reaction was refluxed for 72 hours. To quench, the reaction was cooled to 0 °C, prior to a slow addition of 1 M NaOH. The quenched mixture was then concentrated under reduced pressure before addition of water and extraction of the aqueous phase with $CHCl_3$. Combined organic phases were dried over $MgSO_4$, the salts filtered and the solvent evaporated under reduced pressure. The brown residue was used without further purification (6.8 g, 60 % yield). *MS TOF ES+: $C_{42}H_{74}N_2$, calculated, 606.59, $[M+H]^+$ found 607.5931.*

Synthesis of *N*,*N'*-(naphthalene-1,5-diyl)bis(2-chloro-*N*-hexadecylacetamide)

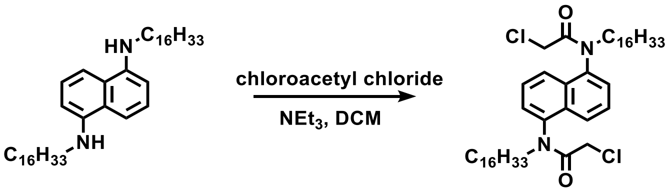

The solution of *N*[1],*N*[5]-dihexadecylnaphthalene-1,5-diamine (3.49 g, 5.6 mmol) in 168 mL DCM was cooled to 0 °C and triethylamine (1.9 mL, 13.4 mmol) injected into the flask. Chloroacetyl chloride was then added dropwise to the reaction. The reaction was allowed to warm to room temperature and quenched with saturated solution of $NaHCO_3$. The phases were separated and the aqueous phases extracted with DCM. The combined organic phases were dried over $MgSO_4$, filtered and the solvent removed under reduced pressure. The brown residue was purified by column chromatography in 1:5 ethyl acetate/petroleum spirit. *^{1}H NMR (400 MHz, $CDCl_3$, 300K): δ= 7.93 (d, J = 8.5 Hz, 2H), 7.74 – 7.62 (m, 2H), 7.53 (d, J = 7.2 Hz, 2H), 4.33 (ddd, J = 13.0, 10.2, 5.9 Hz, 2H), 3.86 – 3.59 (m, 4H), 3.37 – 3.19 (m, 2H), 1.82 – 1.49 (m, 4H), 1.26 (d, J = 6.5 Hz, 52H), 0.90 (t, J = 6.8 Hz, 6H). MS TOF ES+: $C_{46}H_{76}N_2O_2$, calculated, 758.53, $[M+H]^+$ found 759.5391.*

Synthesis of 3,8-dihexadecyl-1,3,6,8-tetrahydroindolo[7,6-g]indole-2,7-dione

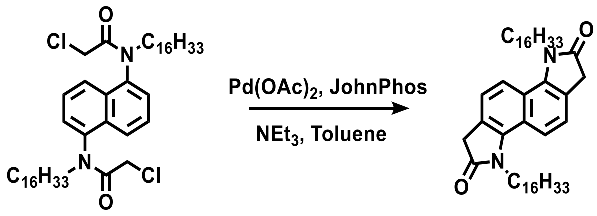

A dry vial is charged with $Pd(OAc)_2$ (53.9 mg, 0.24 mmol, 12 mol %), 2-(di-tert-butylphosphino)biphenyl (JohnPhos, 143.2 mg, 0.48 mmol, 24.0 mol %) and *N*,*N'*-(naphthalene-1,5-diyl)bis(2-chloro-*N*-hexadecylacetamide) (1.52 g, 2.0 mmol). The vial was purged and backfilled with argon three times. Degassed dry toluene (16 mL) was then injected into the flask, followed by triethylamine (0.82 mL, 5.88 mmol). The reaction mixture was then heated to 90 °C for 16 hours. Then the reaction was cooled to room temperature and the solvent removed. The dark residue was purified by flash chromatography with 1:5 ethyl acetate/petroleum spirit 40-60 °C as eluent to furnish off white solid (0.47 g, 34 %). *^{1}H NMR (400 MHz, $CDCl_3$, 300K): δ= 7.95 (d, J = 8.6 Hz, 2H), 7.43 (d, J = 8.6 Hz, 2H), 4.33 – 4.21 (m, 4H), 3.70 (s, 4H), 1.84 (t, J = 7.8 Hz, 4H), 1.27 (s, 50H), 0.98 – 0.83 (m, 6H). MS TOF ES+: $C_{46}H_{74}N_2O_2$, calculated 686.58, $[M+H]^+$ found 687.5873.*

Synthesis of Thienothiophene Core Monomer

Dimethyl thieno[3,2-*b*]thiophene-3,6-dicarboxylate was synthesized according to literature procedures.[4]

Synthesis of Thieno[3,2-*b*]thiophene-3,6-dicarboxylic acid

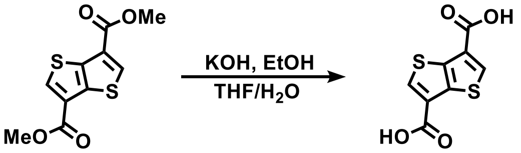

To a mixture of dimethyl thieno[3,2-*b*]thiophene-3,6-dicarboxylate (4.79g, 18.69 mmol, 1.0 e.q.) in ethanol/tetrahydrofuran/water (300 mL/300 mL/30 mL) was added sodium hydroxide (9.33 g, 233.2 mmol, 12.5 e.q). After the reaction mixture was refluxed overnight, the solvent was evaporated under vacuum to about half of its original volume. Water (300 mL) was added to the mixture and the solution was treated with concentrated hydrochloric acid until white precipitates formed. The precipitate was filtered and then washed with water to give thieno[3,2-*b*]thiophene-3,6-dicarboxylic acid as a white solid which was dried in a vacuum oven and then used in the next step without further purifications (3.80 g, 89% yield). *^{1}H NMR (400 MHz, DMSO-d6, 300K): δ= 13.33 (s, 2H), 8.48 (s, 2H). ^{13}C NMR (101 MHz, DMSO-d6, 300K): δ= 163.24, 138.41, 137.69, 126.77.*

Synthesis of Di-*tert*-butyl thieno[3,2-*b*]thiophene-3,6-diyldicarbamate

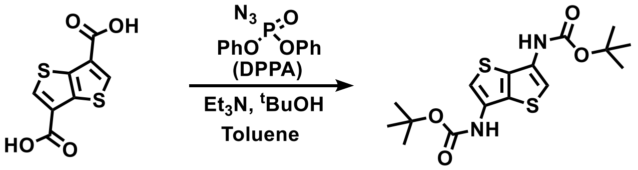

Thieno[3,2-*b*]thiophene-3,6-dicarboxylic acid (3.00 g, 13.14 mmol, 1.0 e.q.), diphenylphosphoryl azide DPPA (6.80 mL, 31.54 mmol, 2.40 e.q.) and triethylamine (4.40 mL, 31.54 mmol, 2.40 e.q.) were combined in anhydrous *tert*-butanol (12.6 mL, 131.40 mmol, 10.0 e.q.) and anhydrous toluene (53 mL). The resulting mixture was heated to reflux. After reacting overnight, the solution was cooled and then concentrated in vacuum to remove solvent. The residue was taken up in diethyl ether and washed with 5 % aqueous citric acid, water and brine, dried with $MgSO_4$, and concentrated. The crude product was purified by silica gel column chromatography with ethyl acetate/ petroleum spirit 40-60 ^{o}C (1:4) as the eluent to give di-*tert*-butyl thieno[3,2-*b*]thiophene-3,6-diyldicarbamate as a light brown solid. (3.51 g, 72% yield). *^{1}H NMR (400 MHz, $CDCl_3$, 300K): δ= 7.38 (s, 2H), 6.60 (s, 2H), 1.57 (s, 18H). ^{13}C NMR (101 MHz,$CDCl_3$, 300K): δ= 152.68, 130.26, 128.90, 108.74, 81.36, 77.41, 77.16, 76.91, 28.42.*

Synthesis of Di-tert-butyl thieno[3,2-*b*]thiophene-3,6-diylbis((2-hexyldecyl)carbamate)

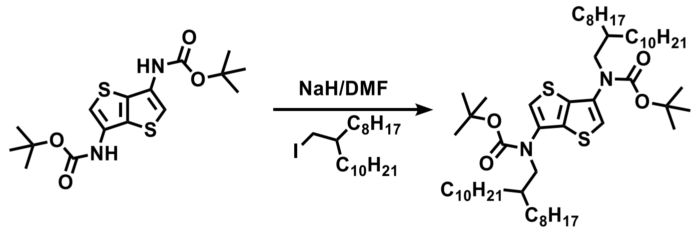

Di-*tert*-butyl thieno[3,2-*b*]thiophene-3,6-diyldicarbamate (2.22 g, 6.0 mmol, 1.0 e.q.) was dissolved in DMF (72 mL) and cooled to 0°C. Sodium hydride (0.96 g, 60% dispersion in mineral oil, 24.0 mmol, 4.0 e.q.) was added and the solution was stirred at room temperature for 1 hour. 9-(Iodomethyl)nonadecane (7.35 g, 18.0 mmol, 3.0 e.q.) was added to the mixture and the solution was stirred at 80 °C for 3 hours. After the solution cooled to room temperature, the mixture was poured into iced water, followed by extraction with ethyl acetate for three times. The organic layers were combined, washed with water, brine and then dried over $MgSO_4$, concentrated. The resulting brown oil was purified via silica gel column chromatography with ethyl acetate/ petroleum spirit 40-60 °C (95:5) as the eluent to give di-tert-butyl thieno[3,2-*b*]thiophene-3,6-diylbis((2-hexyldecyl)carbamate) as a light brown oil (4.9 g, 88% yield). *^{1}H NMR (400 MHz, $CDCl_3$, 300K): δ= 7.04 (s, 2H), 3.62 (d, J = 7.2 Hz, 4H), 1.58-1.48 (m, 2H), 1.34-1.11 (m, 64H), 0.87 (q, J = 6.8 Hz, 12H). ^{13}C NMR (101 MHz, $CDCl_3$, 300K): δ= 154.13, 134.76, 133.70, 119.18, 80.64, 53.38, 36.93, 31.90, 31.77, 31.21, 30.03, 29.68, 29.52, 29.29, 28.15, 26.31, 26.25, 22.66, 22.62, 14.08. MS ESI+: $C_{56}H_{102}N_2O_4S_2$, calculated 930.73, $[M+H]^+$ found 931.53.*

Synthesis of *N*^{3}, *N*^{6}-bis(2-hexyldecyl)thieno[3,2-*b*]thiophene-3,6-diamine

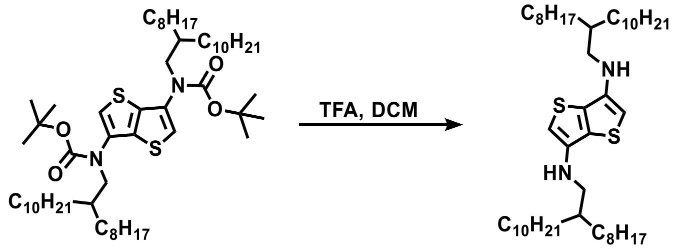

Di-tert-butyl thieno[3,2-*b*]thiophene-3,6-diylbis((2-hexyldecyl)carbamate) (4.66 g, 5.0 mmol, 1.0 e.q.) was dissolved in dichloromethane (100 mL) and cooled to 0 °C. Trifluoroacetic acid (6.5 mL) was added and the reaction mixture was allowed to warm to room temperature and stirred for overnight. The mixture was poured into water, washed with sodium bicarbonate, brine, dried over $MgSO_4$ and then concentrated to afford *N*^{3}, *N*^{6}-bis(2-hexyldecyl)thieno[3,2-*b*]thiophene-3,6-diamine a light brown oil. The product is unstable in air and used immediately without further purifications in the next step (3.25 g, 89% yield). *^{1}H NMR (400 MHz, $CDCl_3$, 300K): δ= 5.97 (s, 2H), 3.53 (s, 2H), 3.10 (d, J = 6.1 Hz, 4H), 1.72-1.63 (m, 2H), 1.50-1.16 (m, 64H), 0.91 (t, J = 6.6 Hz, 12H). ^{13}C NMR (101MHz, $CDCl_3$, 300K): δ= 141.39, 130.01, 94.74, 77.41, 77.16, 76.91, 49.75, 37.82, 32.06, 30.21, 29.83, 29.80, 29.76, 29.51, 29.50, 26.88, 22.85, 14.30.*

Synthesis of Thieno[3,2-*b*]thiophene Bis-isatin

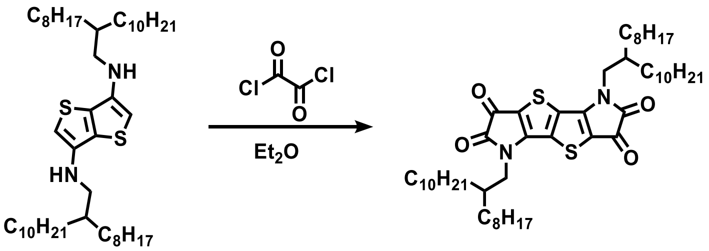

N^{3}, *N*^{6}-bis(2-hexyldecyl)thieno[3,2-*b*]thiophene-3,6-diamine (4.33 g, 5.92 mmol, 1.0 e.q.) in anhydrous diethyl ether (70 mL) was added dropwise to a stirred solution of oxalyl chloride (10.0 mL, 118.4 mmol, 20.0 e.q.) in anhydrous diethyl ether (140 mL) at 0 ^{o}C. The mixture was allowed to warm to room temperature and then stirred for overnight. The mixture was poured into iced water (Caution: gaseous hydrogen chloride produced from oxalyl chloride and water.) and then extracted with ethyl acetate for three times. The organic layers were combined, washed with water, brine and then dried over $MgSO_4$, concentrated. The crude product was purified via silica gel column chromatography with dichloromethane/ petroleum spirit 40-60 ^{o}C (3:2) as the eluent and then recrystallized with dichloromethane/methanol to give the product as a dark green solid (103 mg. 2% yield). *^{1}H NMR (400 MHz, $CDCl_3$, 300K): δ= 3.70 (d, J = 7.7 Hz, 4H), 1.87 (q, J = 6.4 Hz, 2H), 1.47-1.20 (m, 64H), 0.93-0.86 (m, 12H). ^{13}C NMR (101 MHz, $CDCl_3$, 300K): δ= 173.02, 160.00, 157.06, 134.45, 116.98, 47.23, 38.99, 31.86, 31.73, 31.28, 29.97, 29.64, 29.49, 29.28, 26.24, 26.19, 22.67, 22.64, 14.13, 14.09. MS TOF LD+: $C_{50}H_{82}N_2O_4S_2$, calculated 838.5, $[M+H]^+$ found 841.2.*

DFT Calculated Absorption Spectra and Hole and Electron Wavefunctions in the S_1 State

DFT Calculated Torsional Barriers

The torsional potentials between adjacent aromatic units of the various oligomers calculated at the OT-ωB97XD/6-31G(d,p) level of theory.

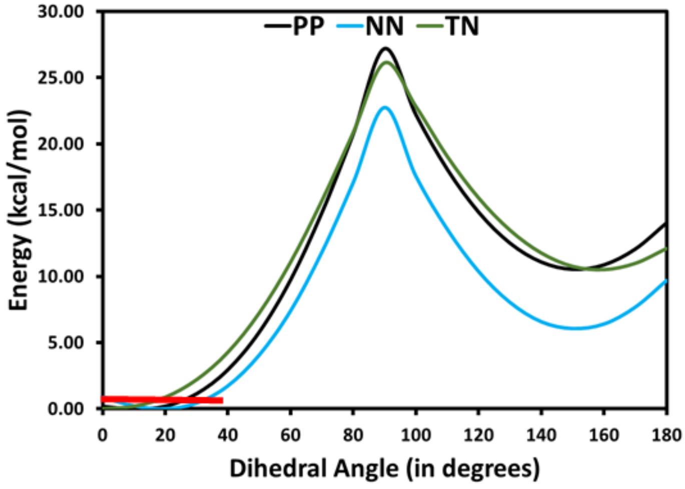

Name	Backbone structure
PP	Phenylene-Phenylene
NN	Naphthalene-Naphthalene
TN	Thienothiophene-Naphthalene

Supplementary Figure 3: Torsional potential profile of the polymers with changing dihedral angles.

A comparison of the torsional potential profiles for the dihedral angle between two adjacent aromatic cores in the PP, NN, and TN oligomer chains. All values are calculated at the OT-ωB97XD/6-31G(d,p) level of theory. The red line represents 1 kT energy (0.6 kcal/mol).

The optimal confirmations appears around 12º, 18º, and 0º for the PP, NN, and TN oligomers, respectively. It is worth stressing that the double-like character of the inter-unit bonds leads to very large barriers to full rotation, on the order of 25 kcal/mol, which is much higher than the values typical of chains with single-like inter-unit bonds (in the range of 2 to 8 kcal/mol).

A closer look of the torsional potentials (from 0º to 30º) between adjacent aromatic units of the oligomer series to highlight the smoothness of PES around the local minima.

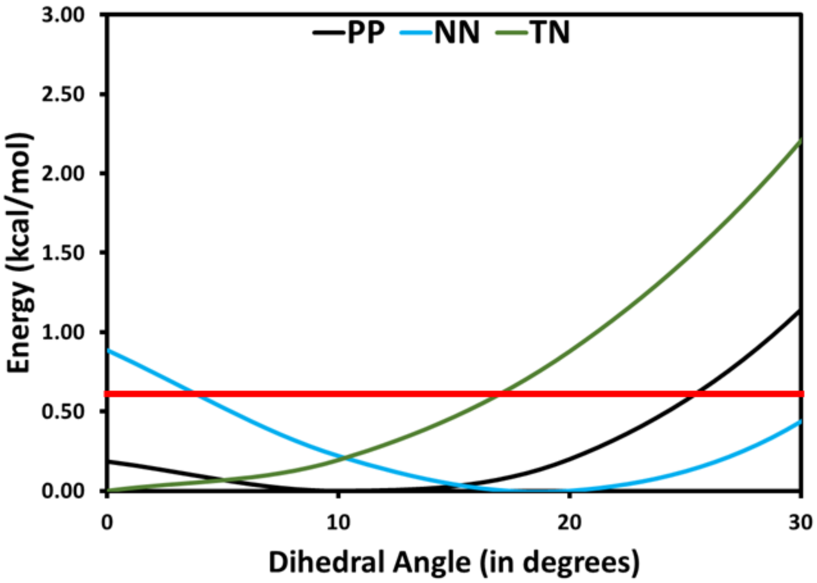

Supplementary Figure 4: Enlarged torsional potential profile of the polymers with changing dihedral angles.

A closer look of the torsional potential profiles for the dihedral angle (from 0º to 30º) between two adjacent aromatic cores in the PP, NN, and TN oligomer chains. All values are calculated at the OT-ωB97XD/6-31G(d,p) level of theory. The red line represents 1 kT energy (0.6 kcal/mol).

The vertical excited-state energies were evaluated at the TD-DFT OT-ωB97XD/6-31g(d,p) level of theory. The simulated absorption spectra calculated for the isolated oligomers ("in the gas phase") are shown below.

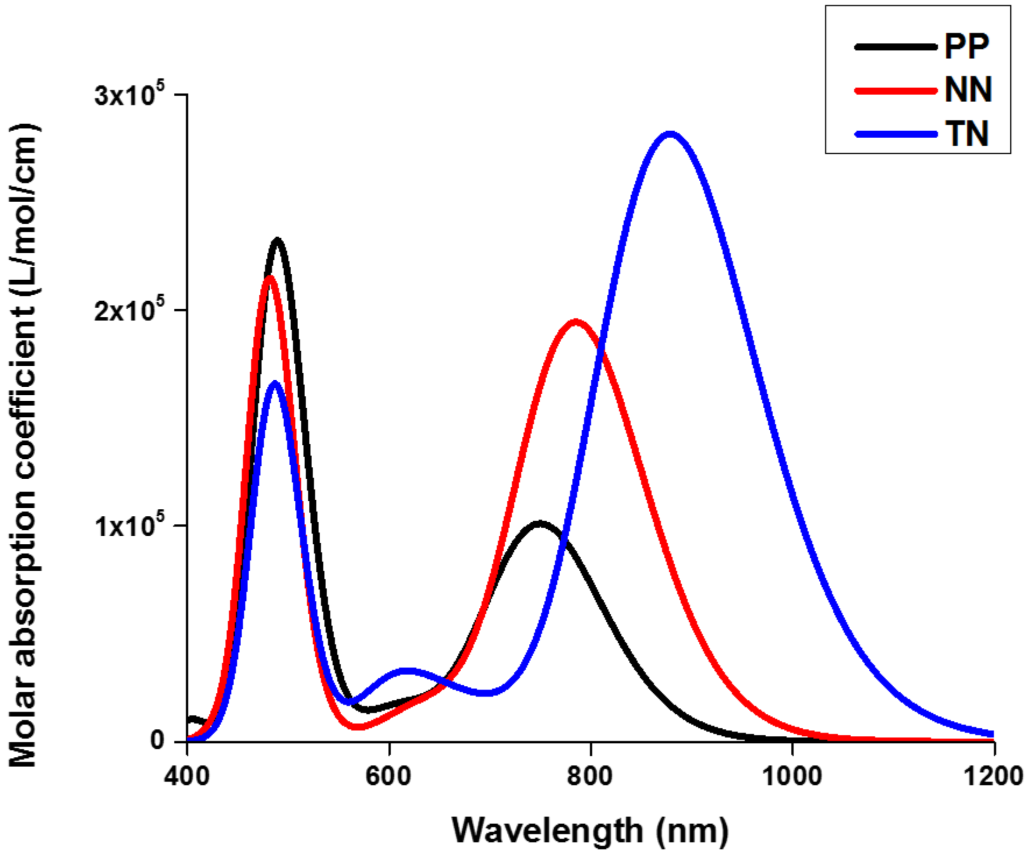

Supplementary Figure 5: **Simulated absorption spectra for the PP, NN, and TN**

The simulated absorption spectra of isolated oligomers were calculated at the TD-OT-ωB97XD/6-31G(d,p) level of theory. The spectra are simulated based on a Gaussian-function convolution (FWHM=0.3 eV) of the oligomer vertical excitation energies and oscillator strengths.

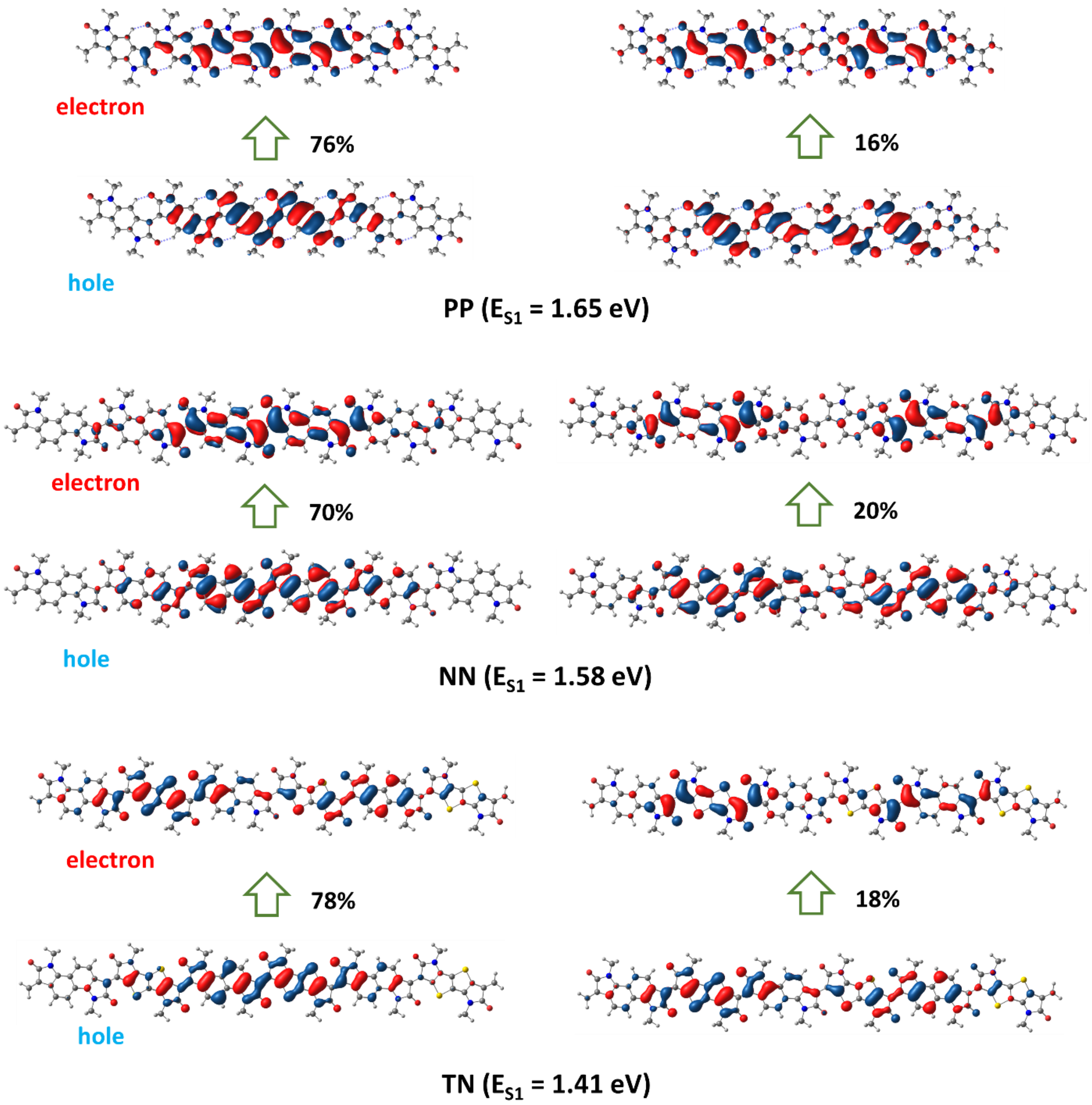

Supplementary Figure 6: Calculated hole and electron wavefunction of PP, NN, TN

Illustration of the hole and electron wavefunctions (isovalues 0.02 a.u.) determined at the TD-OT-ωB97XD/6-31G(d,p) level of theory for the S_0 to S_1 transitions in the PP, NN, and TN oligomers. The contributions of the various hole-electron configurations are given in percentages.

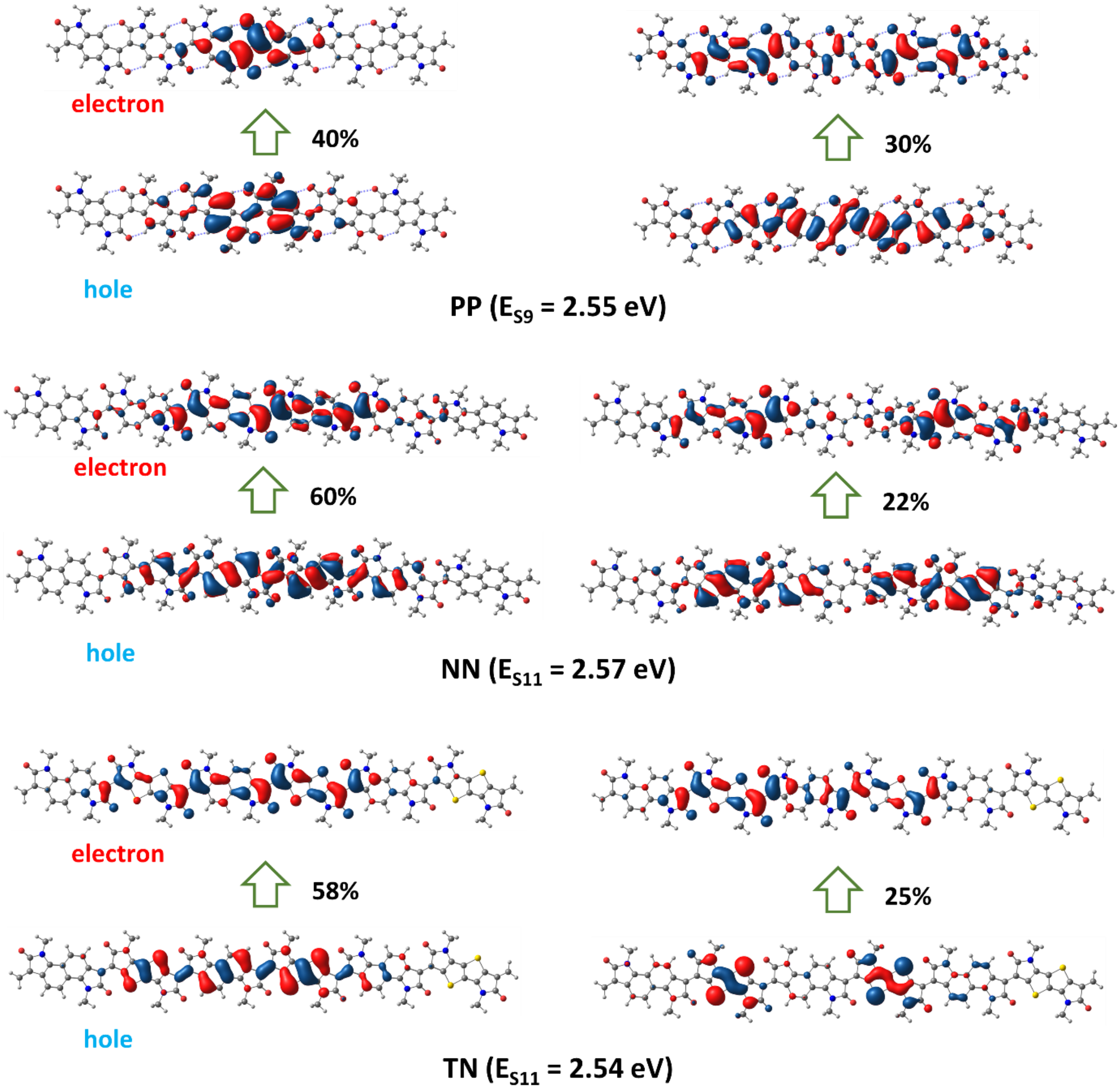

Supplementary Figure 7: Calculated hole and electron wavefunction of PP, NN, TN for S_0 – S_n.

Illustration of the hole and electron wavefunctions (isovalues 0.02 a.u.) determined at the TD-OT-ωB97XD/6-31G(d,p) level of theory for the S_0 to S_n transitions in the PP, NN, and TN oligomers. The contributions of the various hole-electron configurations are given in percentages.

Supplementary Table 1: The simulated oligomer reorganisation energy, ionisation potential and electron.

Ionization potentials and electron affinities of the isolated oligomers calculated at the tuned-ωB97XD/6-31G(d,p) level of theory. Reorganization energies in the anionic state of the oligomers, calculated at the tuned ωB97XD/6-31G(d,p) level of theory.

	IP (eV)	EA (eV)	Reorganization energy (meV)
PP	6.25	2.47	427
TN	5.67	2.57	374
NN	6.00	2.44	327

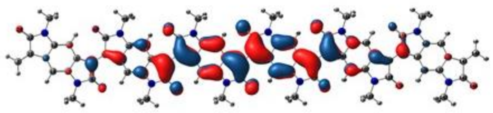

PP

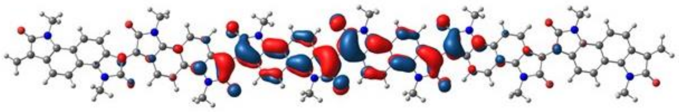

NN

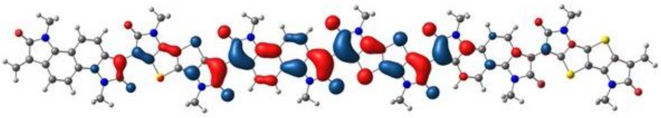

TN

Supplementary Figure 8: Structures of the anionic oligomer chains and polaron wavefunctions.

The anionic oligomer chains and polaron wavefunctions (isovalues of 0.02 electrons$^{1/2}$/bohr$^{3/2}$) were obtained at tuned ωB97XD/6-31G(d,p) level of theory.

UV-Vis and NIR Spectra of Polymers

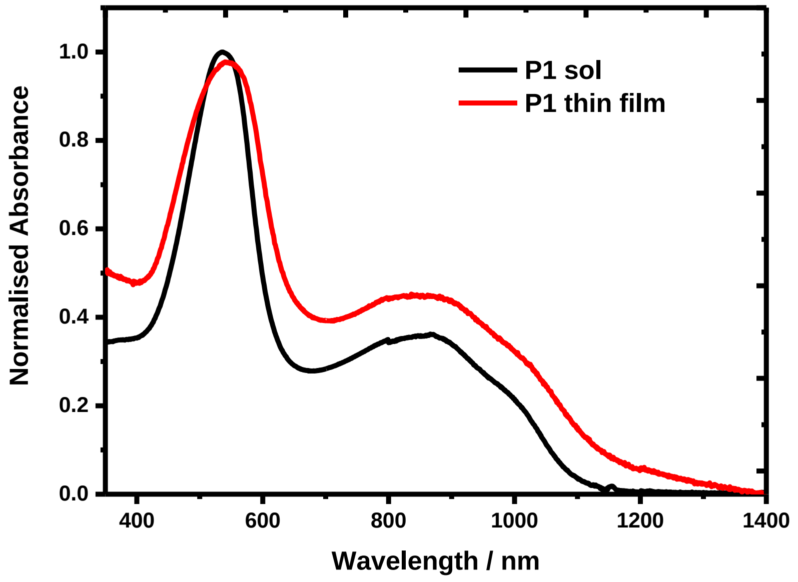

Supplementary Figure 9: UV-Vis near infra-red absorption spectra of P1.

Normalised UV-Vis NIR spectra of polymer **P1** in chlorobenzene and thin film spun from chlorobenzene 5 mg/mL.

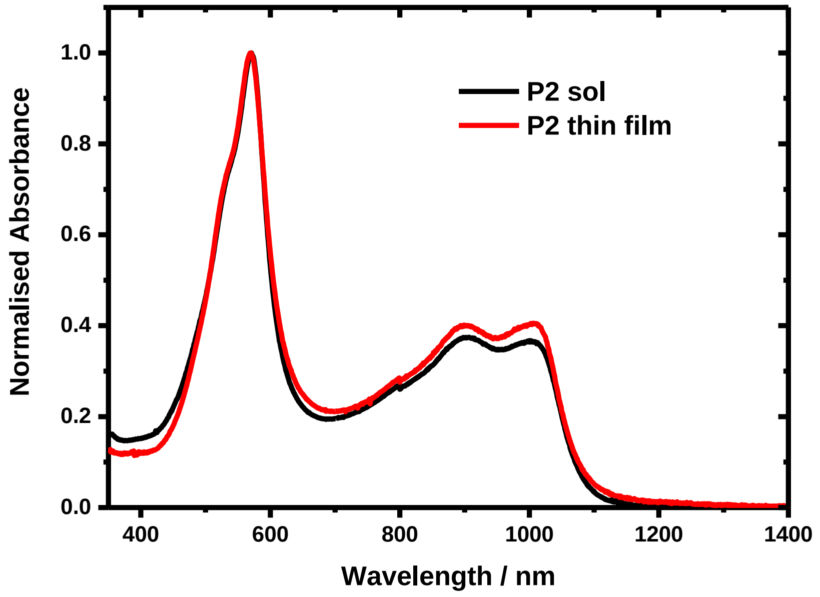

Supplementary Figure 10: UV-Vis near infra-red absorption spectra of P2.

Normalised UV-Vis NIR spectra of polymer **P2** in chlorobenzene and thin film spun from chlorobenzene 5 mg/mL.

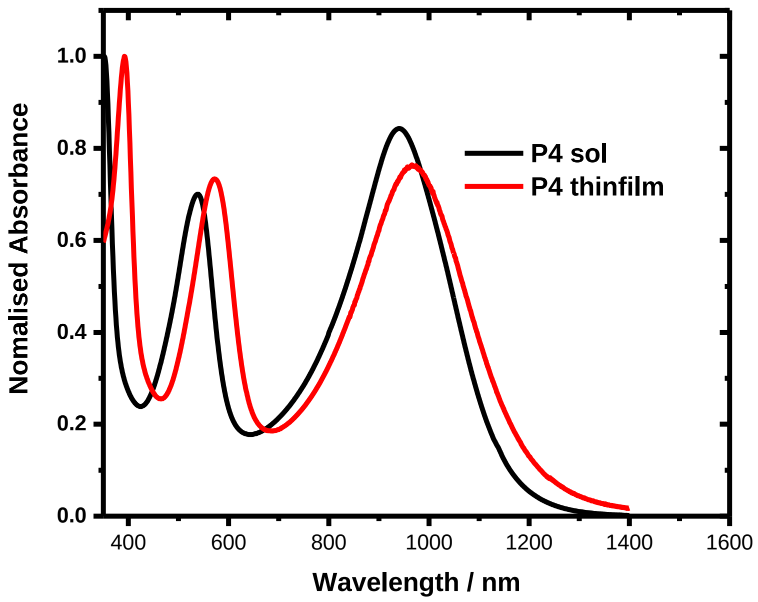

Supplementary Figure 11: UV-Vis near infra-red absorption spectra of P4.

Normalised UV-Vis NIR spectra of polymer **P4** in chlorobenzene and thin film spun from chlorobenzene 5 mg/mL.

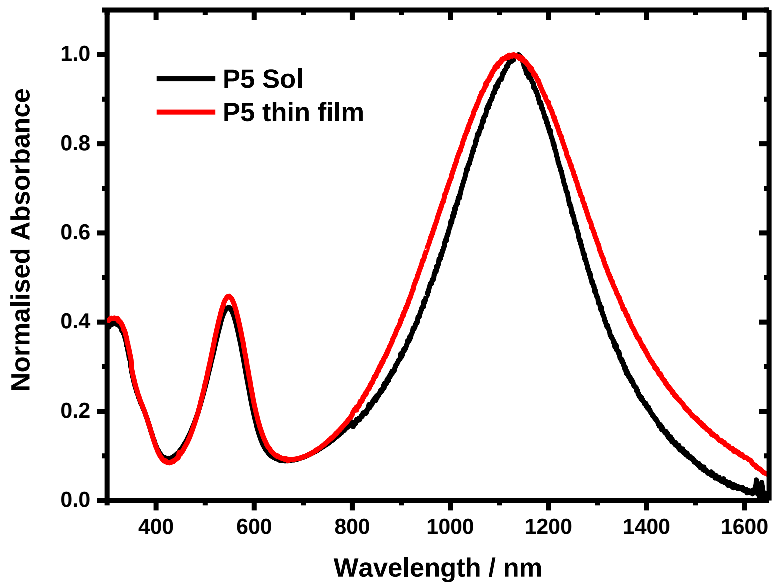

Supplementary Figure 12: UV-Vis near infra-red absorption spectra of P5.

Normalised UV-Vis NIR spectra of polymer **P5** in chlorobenzene and thin film spun from chlorobenzene 5 mg/mL.

Temperature Dependent and Solvatochromic Studies on the Data

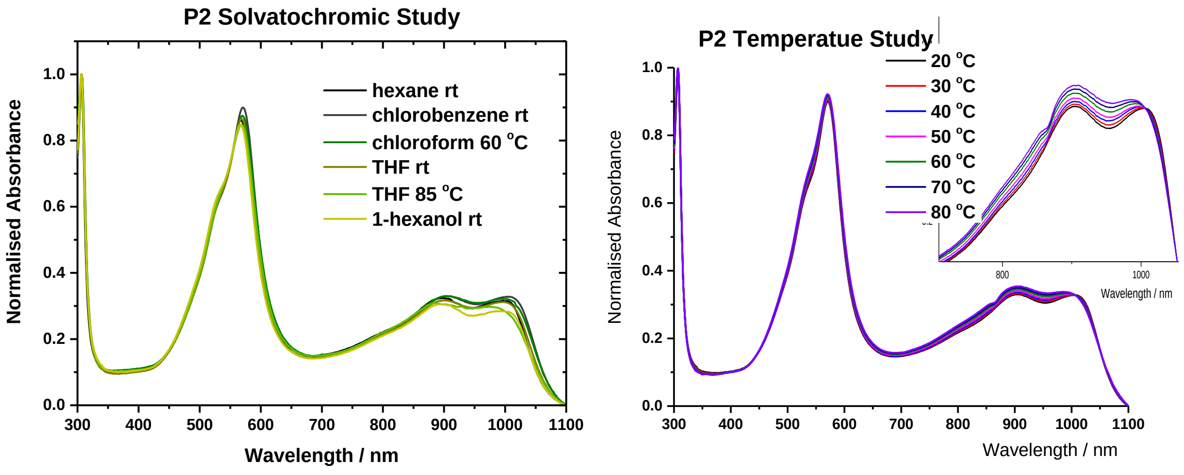

Supplementary Figure 13: Solvatochromic and temperature studies of polymer P2

There is insignificant changes to the absorbance upon heating from 20- 90 °C. A slight blue shift, as excepted, is observed. This polymer was measured because it has mobility of 0.03 $cm^2V^{-1}s^{-1}$.

GIWAX Scattering of the Polymers

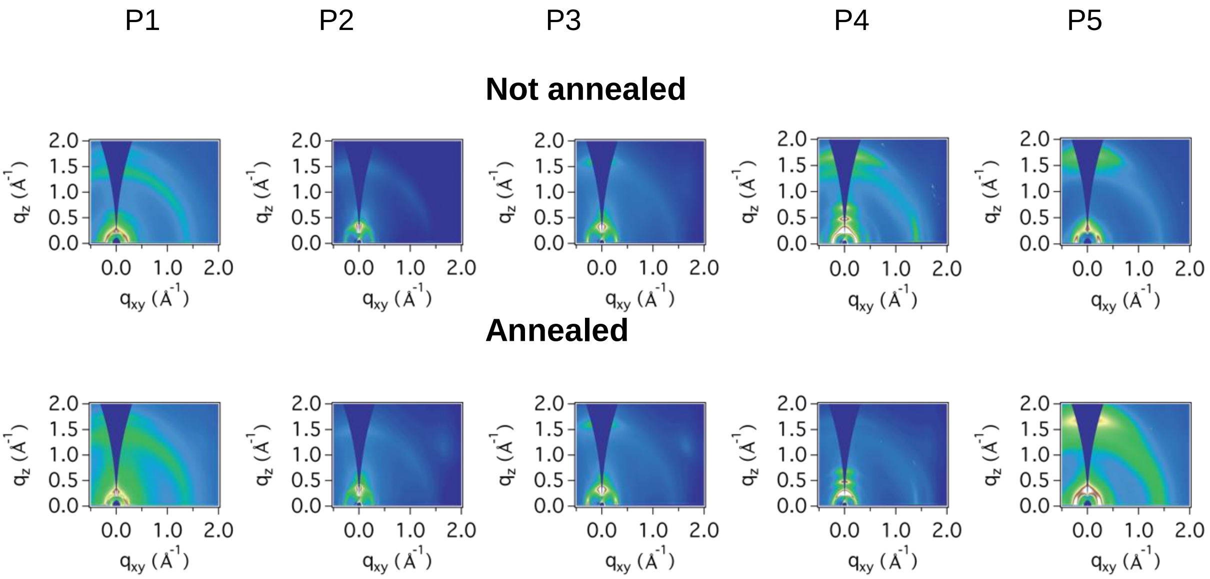

Supplementary Figure 14: Grazing incidence wide angle X-ray scattering of the polymers

Grazing incidence wide angle X-ray scattering (GIWAXS) of polymer P1, P2, P3, P4 and P5 film, spun at room temperature (left) and annealed at 200 °C (right).

Polymer Synthesis

Synthesis of Polymer P1

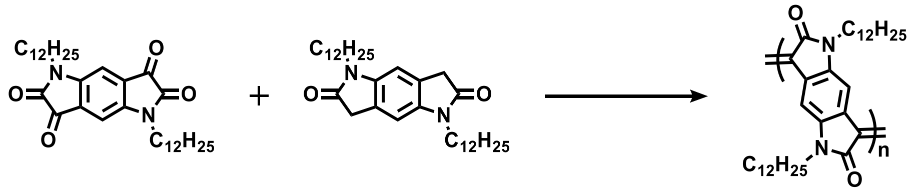

P1

A microwave vial was charged with 1,5-didodecyl-1,5-dihydropyrrolo[2,3-f]indole-2,3,6,7-tetraone (50.0 mg, 0.06 mmol), 1,5-didodecyl-5,7-dihydropyrrolo[2,3-f]indole-2,6(1H,3H)-dione (51.6 mg, 0.06 mmol), p-toluene sulfonic acid monohydrate (3.3 mg, 0.02 mmol). The vial was sealed and dry degassed toluene (2 mL) injected into the vial. The reaction was heated at 120 ^{o}C for 21 hours followed by 10 hours at 140 ^{o}C. The reaction mixture changed colour from blue to red/brown to dark purple over the polymerisation period. The crude polymer was precipitated in methanol and purified by Soxhlet extraction with methanol, acetone, and hexane dichloromethane and chloroform. The hexane fraction was collected and reduced under vacuum and the polymer precipitated into methanol. The polymer was filtered and dried. Yield: 83 mg, 82 % dark purple solid. GPC (chlorobenzene, 80 °C): Mn 14kDa, Mw 19kDa.

Synthesis of Polymer P2

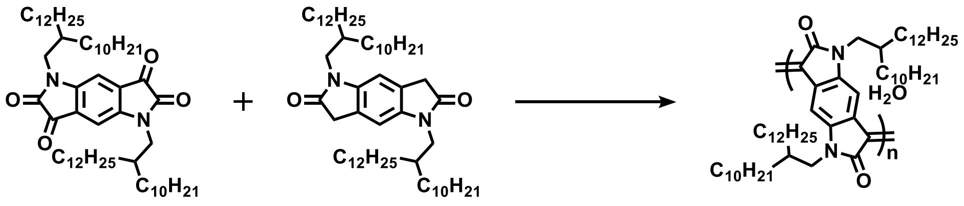

P2

A microwave vial was charged with 1,5-bis(2-decyltetradecyl)-5,7-dihydropyrrolo[2,3-*f*]indole-2,6(1*H*,3*H*)-dione (96.9 mg, 0.11 mmol), 1,5-bis(2-decyltetradecyl)-1,5-dihydropyrrolo[2,3-f]indole-2,3,6,7-tetraone (100.0 mg, 0.11 mmol) and p-toluene sulfonic acid monohydrate (6.5 mg, 0.03 mmol). Dry toluene (2 mL), already degassed for 30 minutes was injected into the sealed vial. The reaction was heated at 140 ^{o}C for 48 hours. The reaction mixture changed colour from blue to red/brown to dark purple over the polymerisation period. The crude polymer was precipitated in methanol and purified by Soxhlet extraction with methanol, acetone, and hexane. The hexane fraction was collected and reduced under vacuum and the polymer precipitated into methanol. The polymer was filtered and dried. Yield: 171 mg, 87 % dark purple solid. *^{1}H NMR (400 MHz, CDCl3, 300K): δ= 9.30 (d, J = 8.2 Hz, 2H), 8.93 (s, 2H), 7.11 (d, J = 8.2 Hz, 2H), 3.75 (dd, J = 13.6, 7.3 Hz, 8H), 2.02 (dt, J = 12.8, 6.1 Hz, 4H), 1.66 – 1.12 (m, 150H), 0.91 – 0.79 (m, J = 3.7 Hz, 24H).* GPC (chlorobenzene, 80 °C): Mn 58kDa, Mw 131kDa.

a)

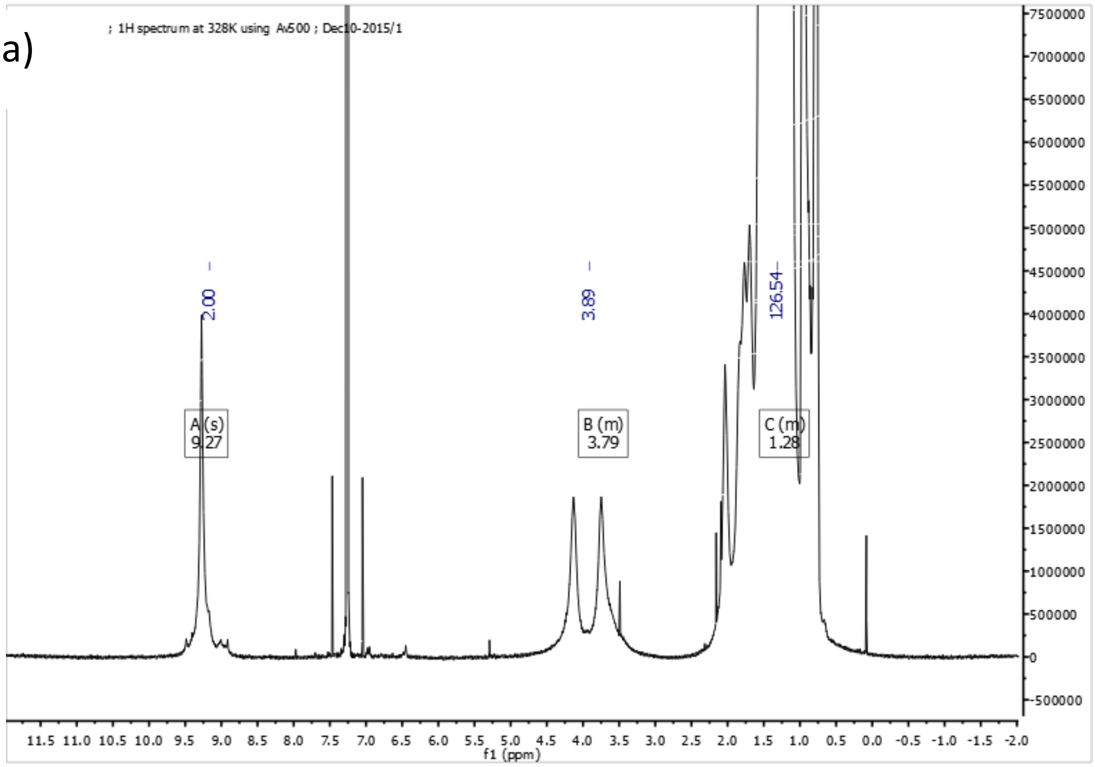

b)

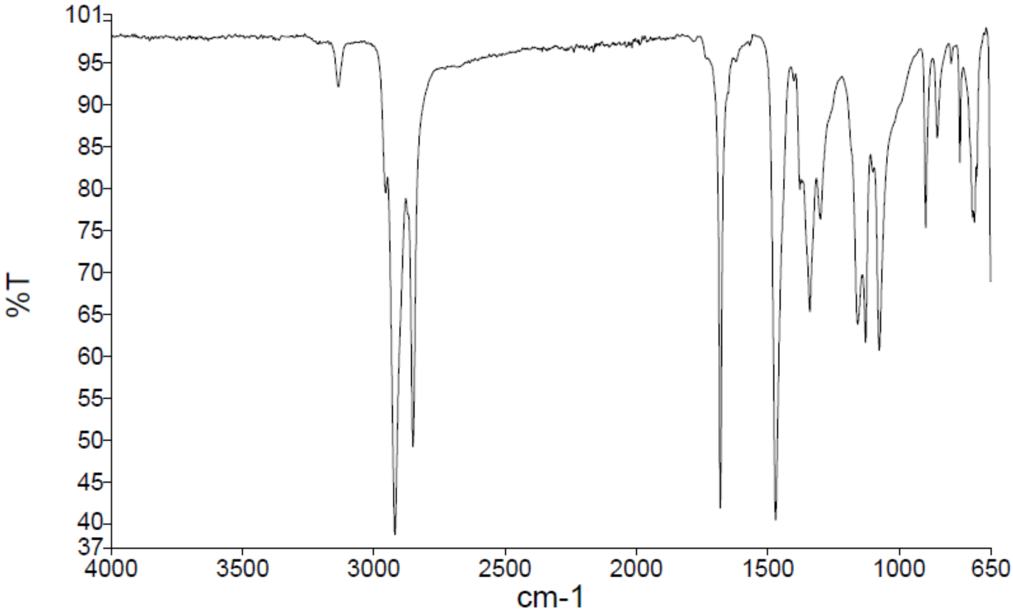

Supplementary Figure 14: NMR and IR spectra of polymer P2

a) Proton NMR spectrum of polymer **P2** in deuterated chloroform at 400 Hz. The signal at 9.27 ppm is from the highly deshielded aromatic proton in the phenyl ring in the polymer. b) The FT-IR spectra of **P2.** There is no hydroxyl absorptions presence suggesting the absence of the aldol condensation intermediates and hydroxy deformations along the polymer backbone.

Synthesis of Polymer P2

A microwave vial was charged with 1,5-bis(2-decyltetradecyl)-5,7-dihydropyrrolo[2,3-f]indole-2,6(1H,3H)-dione (8) (50.0 mg, 0.06 mmol), 1,5-bis(2-decyltetradecyl)-1,5-dihydropyrrolo[2,3-f]indole-2,3,6,7-tetraone (9) (51.6 mg, 0.06 mmol), p-toluene sulfonic acid monohydrate (3.3 mg, 0.02 mmol). The vial was sealed and dry toluene (2 ml), already degassed for 30 minutes was injected into the vial. The reaction was heated at 120 ^{o}C for 31 hours. The reaction mixture changed colour from blue to red/brown to dark purple over the polymerisation period. The crude polymer was precipitated in methanol and purified by Soxhlet extraction with methanol, acetone, and hexane. The hexane fraction was collected and reduced under vacuum and the polymer precipitated into methanol. The polymer was filtered and dried. Yield: 83 mg, 82 % dark purple solid. GPC (chlorobenzene, 80 °C): Mn 18kDa, Mw 30kDa.

Supplementary Table 2: GPC results of P2 at different concentrations

Polymer	Mn(kDa)	Mw(kDa)	PDI	Concentration[a]
**P2**	18	30	1.6	0.030M
**P2**	58	131	2.3	0.055M

a) The concentration was calculated from the molar of one monomer and the volume of the solvent. ($C = n_{monomer}/V_{solvent}$)

Synthesis of Polymer P3

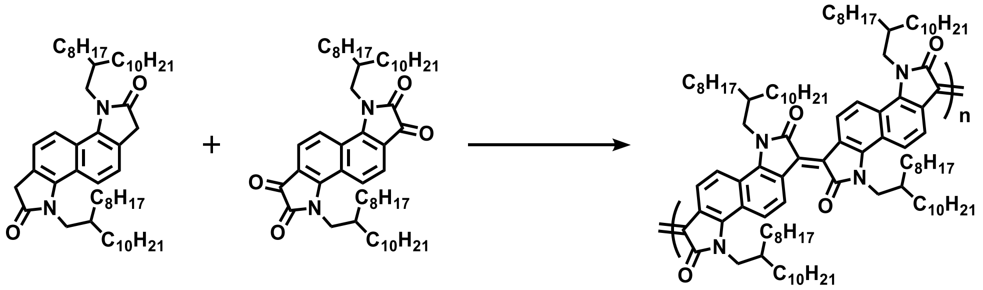

(N-(2-octyltetradecyl))-napthalene bisoxindole (41.80 mg, 0.0523mmol), (N-(2-octyltetradecyl))-napthalene bisisatin (43.26mg, 0.0523mmol) and PTSA monohydrate (4 mg, 0.3eq) were placed into a dry 2mL microwave vial which was capped and evacuated with argon. 0.5mL of degassed dry toluene was added and the mixture was heated at 120°C for 2 hours to give a purple solid. Chlorobenzene was added and the polymer was precipitated into methanol. Successive soxhlet extractions with methanol, acetone, hexane and finally DCM gave a single major polymer fraction, which was reduced to minimum volume and precipitated into methanol to give 47 mg of a purple solid (56%yield). GPC (chlorobenzene, 80 °C): Mn 214 kDa, Mw 677 kDa.

Synthesis of Polymer P4

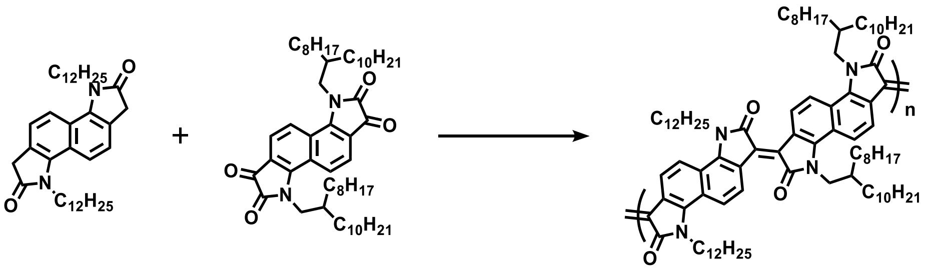

3,8-didodecyl-3,8-dihydroindolo[7,6-*g*]indole-1,2,6,7-tetraone (66.37mg, 0.08 mmol), 3,8-didodecyl-1,3,6,8-tetrahydroindolo[7,6-*g*]indole-2,7-dione (46.12 mg, 0.08 mmol) and p-toluene sulfonic acid monohydrate (4.0 mg, 0.02 mmol) were loaded into a dry vial and the vial purged and backfilled with argon three times. Pre-degassed anhydrous toluene (1.5 mL) was injected into the flask and heated at 120 °C for 12 hours, Soxhlet with chlorobenzene gave purple polymer 102 mg, 92 % yield. GPC (chlorobenzene, 80 °C): Mn 134 kDa, Mw 538 kDa. ^{1}H NMR (d2-TCE, 403K, 400Hz): δ= 9.36-8.94 ppm (broad), 8.13-7.44 ppm (broad), 4.77-4.03 ppm (broad), 2.44-0.52 ppm (broad).

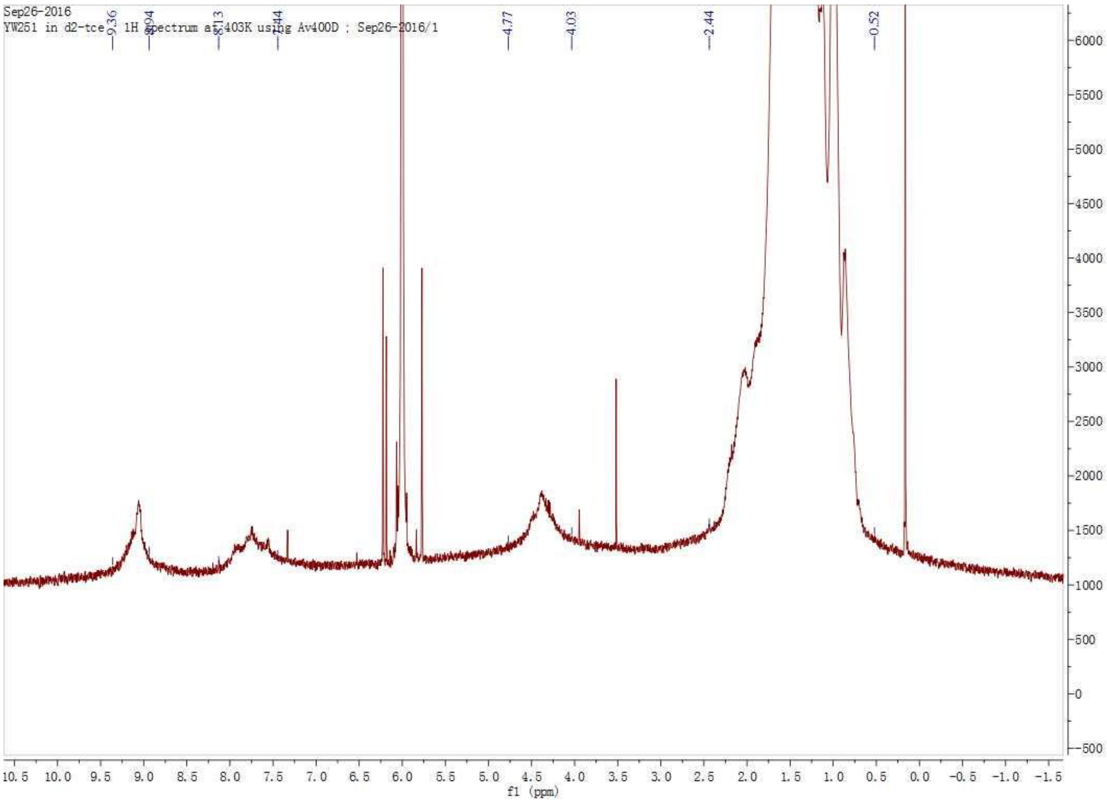

Supplementary Figure 15: NMR spectra of polymer P4

^{1}H NMR of polymer **P4** in d_2-TCE at 403 K.

Synthesis of Polymer P5

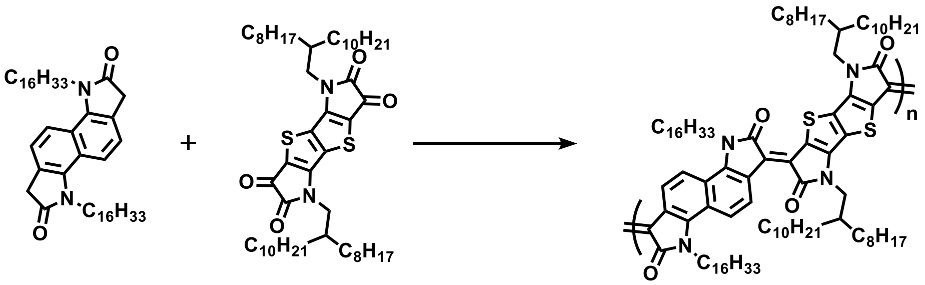

A microwave vial was charged with 1,5-bis(2-octyldodecyl)pyrrolo[2'',3'':4',5']thieno[2',3':4,5]thieno[3,2-b]pyrrole-2,3,6,7(1H,5H)-tetrone (30 mg, 0.04 mmol), 3,8-dihexadecyl-1,3,6,8-tetrahydroindolo[7,6-g]indole-2,7-dione (24.56 mg, 0.04 mmol), p-toluene sulfonic acid monohydrate (2.0 mg, 0.01 mmol). The vial was sealed and dry degassed toluene (0.53 mL) injected into the vial. The reaction was heated at 120 ^{o}C for 16 hours followed by 16 hours at 140 ^{o}C. The crude polymer was precipitated in methanol and purified by Soxhlet extraction with methanol, acetone, and hexane dichloromethane and chloroform. The dichloromethane and chloroform fractions were collected and reduced under vacuum and the polymer precipitated into methanol to give 25 mg and 10 mg respectively. The polymer was filtered and dried. Yield: 35 mg, 66 % dark purple solid. GPC (chlorobenzene, 80 °C): Mn 8.3 kDa, Mw 9.0 kDa.

Gel-Permeation Chromatogram and Thermogravimetric Analysis of Polymers

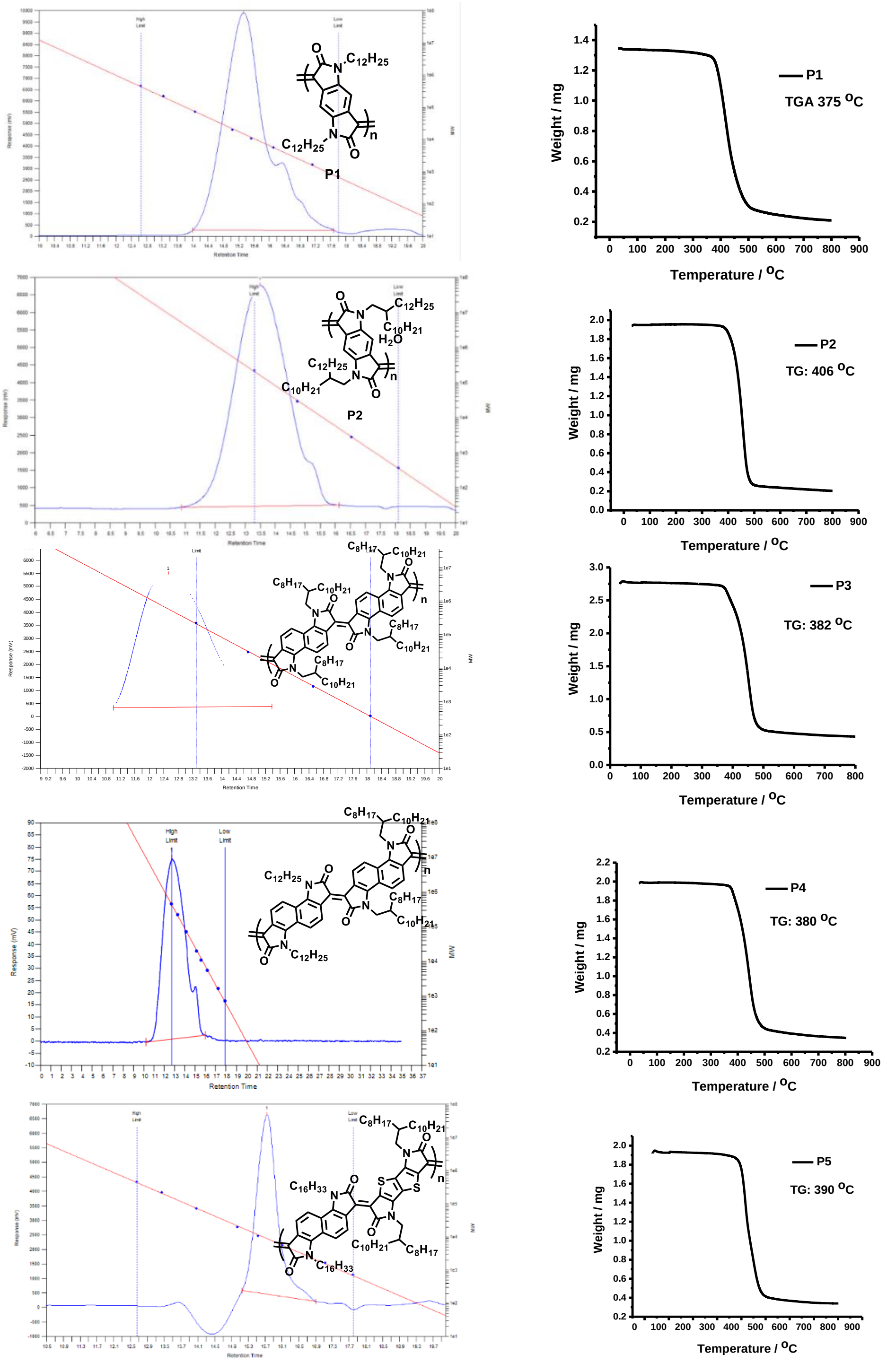

Supplementary Figure 16: GPC and TGA curves for the polymers.

GPC (Mn, Mw, and PDI (Mw / Mn) determined by GPC using low-D (<1.10) polystyrene standards and chlorobenzene as the eluent at 80 °C) and TGA trace (recorded at 10℃/min in a nitrogen atmosphere.) for the polymer series **P1**, **P2**, **P3**, **P4** and **P5**.

Transistor Data

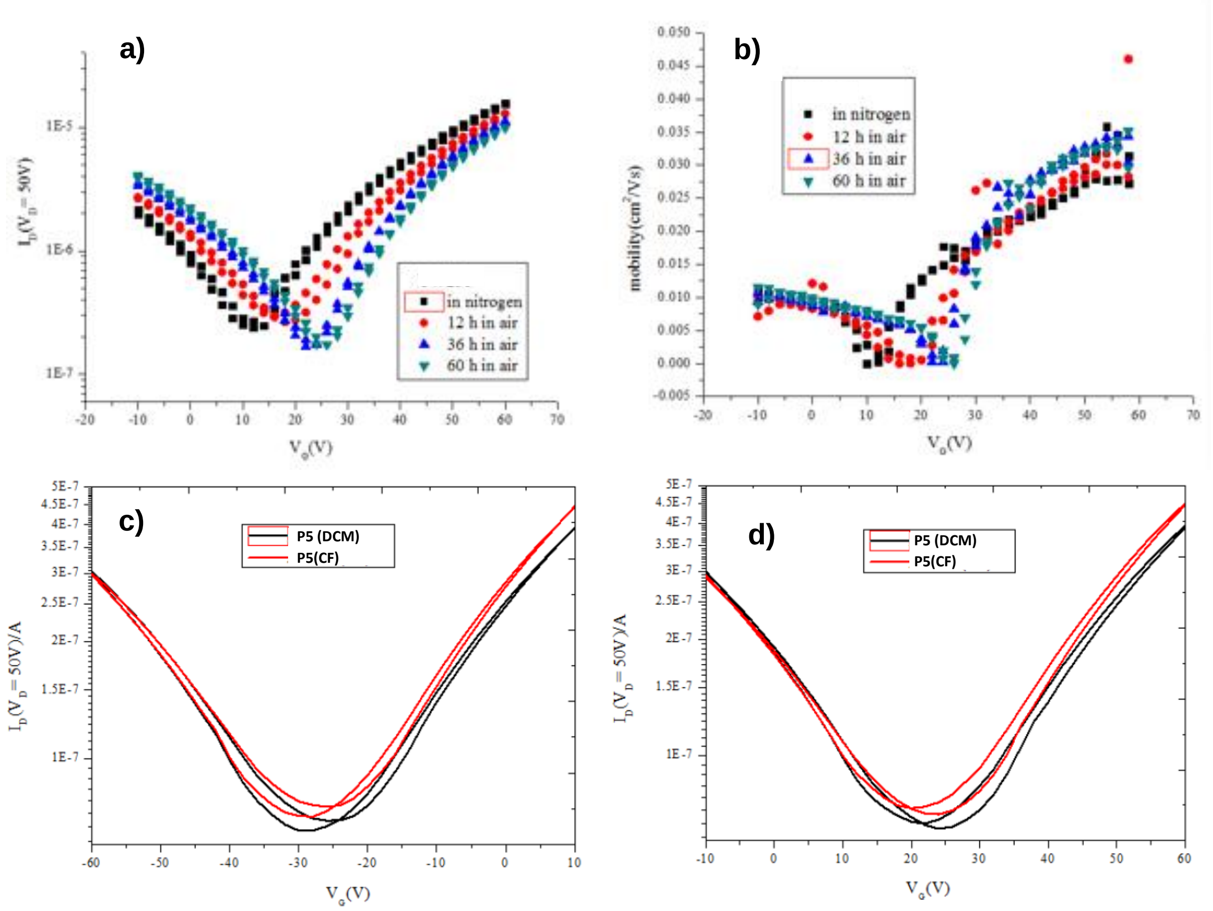

Supplementary Figure 17: Transistor curves for P4 and P5.

Transistor transfer curves for **P4 (a, b)** and **P5 (c,d)**, which has electron charge mobility of 0.03 cm^2/Vs and 0.001 cm^2/Vs respectively.

Supplementary References

1. a) W. Cui, F. Wudl, *Macromolecules* **2013**, *46*, 7232-7238; b) J. W. Rumer, S. Y. Dai, M. Levick, L. Biniek, D. J. Procter, I. McCulloch, J. Polym. Sci., Part A: Polym. Chem. **2013**, *51*, 1285-1291.
2. N. M. Randell, P. C. Boutin, T. L. Kelly, *J. Mater. Chem. A.* **2016**. *4*, 6940-6945.
3. Y. Jiang, Y. Gao, H. Tian, J. Ding, D. Yan, Y. Geng, F. Wang, *Macromolecules*, **2016,** 49, 2135−2144.
4. M. Saito, I. Osaka, Y. Suda, H. Yoshida, K. Takimiya, *Adv. Mater.* **2016**, *28*, 6921–6925.
